# HO-1 Nuclear Interactome Implications in Neuroendocrine Transdifferentiation in Prostate Cancer

**DOI:** 10.3390/ijms27177635

**Published:** 2026-08-26

**Authors:** Rocio Seniuk, Pablo Sanchis, Agustina Sabater, Gaston Pascual, Juan Bizzotto, Julia Lechuga, Magdalena Delfino, Peter D. A. Shepherd, Jiabin Dong, María Pia Valacco, Javier Cotignola, Elba Vazquez, Ayelen Toro, Geraldine Gueron, Estefania Labanca

**Affiliations:** 1 Departamento de Química Biológica, Facultad de Ciencias Exactas y Naturales, Universidad de Buenos Aires, Buenos Aires C1428EGA, Argentina; rseniuk@mdanderson.org (R.S.); pabloasanchis@gmail.com (P.S.); asabater1@mdanderson.org (A.S.); magdadelfino71@gmail.com (M.D.); ggueron@iquibicen.fcen.uba.ar (G.G.); 2 Instituto de Química Biológica de la Facultad de Ciencias Exactas y Naturales (IQUIBICEN), CONICET-Universidad de Buenos Aires, Buenos Aires C1428EGA, Argentina; 3 Department of Genitourinary Medical Oncology and the David H. Koch Center for Applied Research of Genitourinary Cancers, The University of Texas MD Anderson Cancer Center, Houston, TX 77030, USA; pshepherd@mdanderson.org (P.D.A.S.);; 4 Instituto de Tecnología (INTEC), Universidad Argentina de la Empresa (UADE), Buenos Aires C1073AAO, Argentina

**Keywords:** prostate cancer, heme-oxygenase 1, nuclear interactome, neuroendocrine transdifferentiation

## Abstract

Neuroendocrine prostate cancer (NEPC) is characterized by androgen receptor (AR) independence and poor response to conventional therapies, highlighting the need to unveil the molecular mechanisms behind NEPC. We previously showed that heme oxygenase 1 (HO-1, encoded by HMOX1) translocates to the nucleus exerting unexplored non-canonical functions. The present study delineates the nuclear interactome of HO-1, revealing a potential mechanism that impairs NEPC establishment. Through a proteomics approach integrated with bioinformatics analyses, we identified eleven novel nuclear interactors of HO-1. Unsupervised clustering analyses of RNA-seq data from prostate cancer (PCa) patient-derived xenografts (MDA PCa PDXs) and clinical cohorts demonstrated high expression of three HO-1 interactors (ILF3, SAFB, BCLAF1, and DDX17) in NEPC samples, with concomitant HMOX1 under-expression. Spatial transcriptomics in a mixed-histology tumor confirmed enrichment of the interactors in the NEPC foci. To better understand the link between HO-1 and NEPC, we established and characterized an in vitro model of NE transdifferentiation in PCa cell lines using forskolin to induce phenotypic reprogramming. HO-1 upregulation in transdifferentiated cells significantly attenuated the expression of NE markers and triggered a morphological shift, restoring epithelial phenotypes. This work identifies for the first time HO-1 nuclear interactome association with NEPC, where the HMOX1-ILF3-SAFB-BCLAF1-DDX17 print emerges as a useful marker for disease stratification.

## 1. Introduction

Prostate cancer (PCa) ranks as the second most prevalent malignancy among men worldwide and represents the fifth leading cause of cancer-related mortality in this population [1]. PCa exhibits considerable histological heterogeneity, encompassing subtypes such as neuroendocrine (NE), squamous cell, and ductal carcinomas, each characterized by distinct biological features and prognoses [2]. This heterogeneity is mirrored in its clinical course, ranging from indolent forms to highly aggressive tumors, particularly in advanced stages where treatment resistance is prevalent [2].

Neuroendocrine prostate cancer (NEPC) stands out as a particularly aggressive subtype of PCa, defined by androgen receptor (AR) independence and poor prognosis, with median survival often less than one year [3]. NEPC can arise either de novo (0.5–1% of cases) or, more frequently, as a treatment-induced transformation (t-NEPC) occurring in approximately 17% of castration-resistant prostate cancer (CRPC) cases, particularly after prolonged exposure to potent androgen receptor signaling inhibitors such as enzalutamide or abiraterone [4]. NEPC is distinguished by loss of AR expression, low serum PSA levels, and upregulation of NE markers including chromogranin A (CHGA), synaptophysin (SYP), and CD56 (encoded by NCAM1) [3]. Despite the widespread use of canonical neuroendocrine (NE) markers for the histological diagnosis of NEPC and ongoing efforts to identify novel markers capable of better stratifying NEPC subtypes, including ASCL1 [5], NEUROD1 [6], and DLL3 [7], there remains an urgent need for additional biomarkers that enable more accurate and sensitive detection of NEPC.

Given that chronic inflammation is one of the first hallmarks of cancer [8], targeting inflammation-associated molecular pathways is one of many therapeutic strategies to counteract tumor development and progression. In this context, Heme oxygenase-1 (HO-1), encoded by the HMOX1 gene, is a central homeostatic inducible microsomal enzyme that exerts anti-inflammatory, anti-apoptotic, antioxidant, and immunomodulatory effects and ferroptosis-related lipid peroxidation, mediated by heme catabolism byproducts [9]. However, the role of HO-1 in tumor progression remains controversial and context-dependent. Its protumoral or antitumoral nature most likely depends on tumor type and cellular genetic background [10].

Studies from our research group have consistently demonstrated an antitumoral role of HO-1 in PCa [11,12]. HO-1 overexpression in androgen-sensitive and androgen-insensitive PCa cell lines was associated with reduced proliferation, migration, and invasion in vitro, effects that are independent of its enzymatic activity [12]. Moreover, human PCa cell lines with stable HMOX1 overexpression inoculated into athymic nude mice exhibited a marked delay in tumor growth compared to controls [12]. We also described a physical interaction between HO-1 and STAT3, resulting in cytoplasmic retention of this transcription factor, thereby inhibiting AR pathway transcriptional activity [13]. We documented for the first time the nuclear localization of HO-1 in treatment-naïve PCa patient samples across various Gleason scores [14]. Although HO-1 has been reported to interact with proteins that can localize to the nucleus in PCa cells, the specific proteins that interact with HO-1 in the nuclear compartment have not been identified until now [15]. Despite emerging evidence of HO-1 nuclear translocation and its potential association with chromatin, HO-1 lacks canonical DNA-binding domains, and direct evidence supporting its function as a bona fide DNA-binding protein remains elusive [16].

Importantly, the nuclear translocation of HO-1 in PCa, induced by stressors or hemin (a heme analogue approved by the FDA for porphyria treatment [17]), highlights its non-canonical functions beyond heme degradation, including gene regulation and modulation of pathways implicated in increased malignant progression (e.g., inflammation-driven transdifferentiation) [18]. In the context of NEPC, where oxidative stress, inflammation, and lineage plasticity drive resistance to androgen-deprivation therapy (ADT) and NE feature acquisition, the antitumoral effects of HO-1 might represent a protective mechanism against such aggressive disease evolution.

In this study, we characterized part of the nuclear HO-1 interactome through an integrative proteomic and bioinformatic approach, revealing a strong association between HO-1-interacting networks and NEPC biology, while enabling subtype-specific stratification of tumor samples. Importantly, pharmacological induction of HO-1 impaired neuroendocrine transdifferentiation of PCa cells in vitro, supporting a functional role for HO-1 in restraining lineage plasticity and NEPC progression.

## 2. Results

### 2.1. Identification of the Nuclear Interactome of HO-1 in PCa Cells

**Figure 1:**
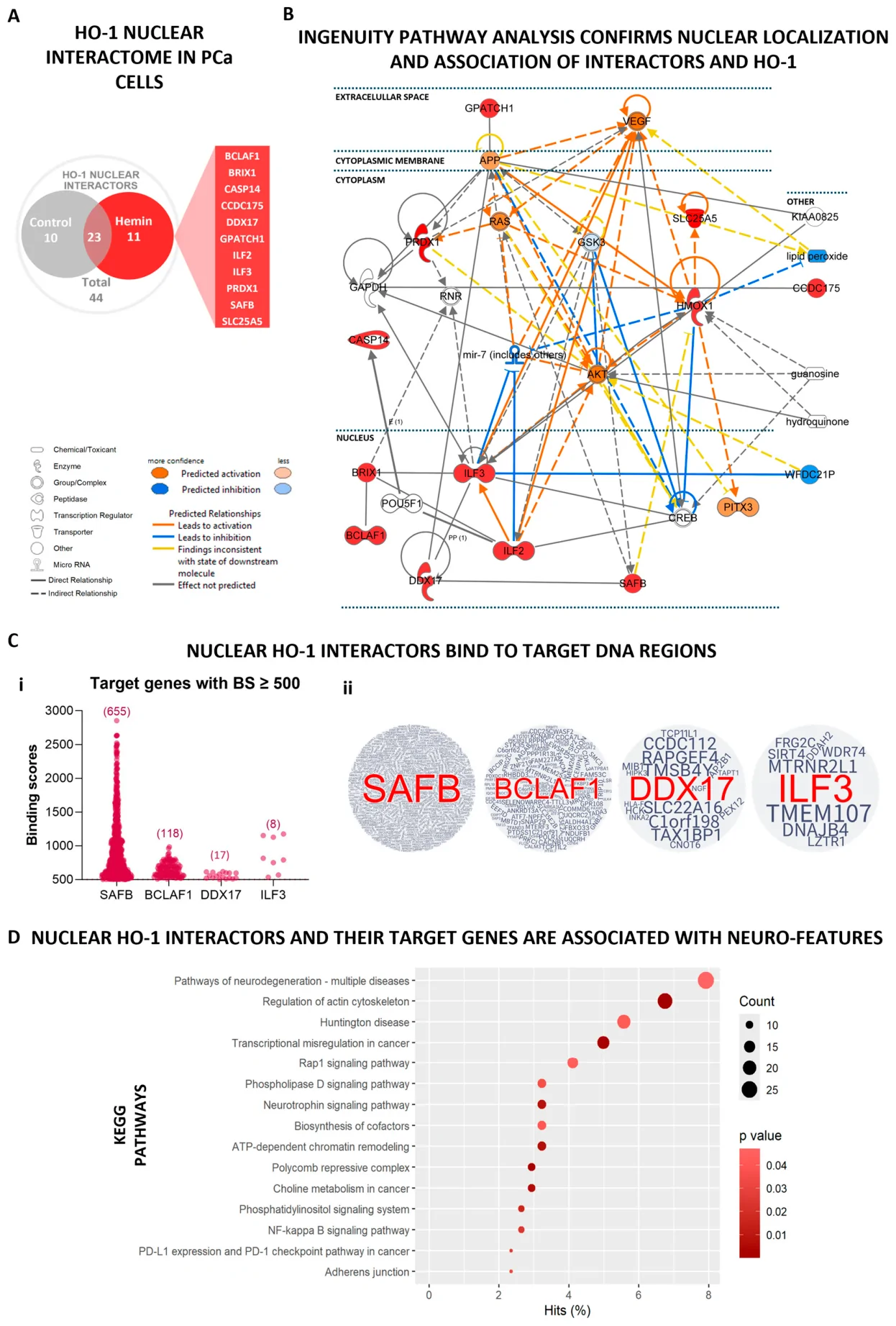
Defining the nuclear interactome of HO-1 and biological implications of downstream targets. (**A**) Venn diagram representing the 44 proteins co-immunoprecipitated with HO-1 and identified by mass spectrometry in the nuclear fraction of PC3 cells, of which 11 (listed) were differentially expressed in cells treated with hemin compared to the control. (**B**) Ingenuity Pathway Analysis (IPA) network using the Peptide-Spectrum Matches (PSMs) from the mass spectrometry analysis. HO-1 interactors are colored in red. (**C**) Analysis of HO-1 interactors using the ChIP-Atlas repository (https://chip-atlas.org/target_genes, accessed on 12 October 2023); (**i**) dot-plot depicting the number of target genes with a binding score of 500 or more for the four HO-1 interactors with DNA-binding sites; (**ii**) word clouds displaying all target genes with a binding score of >500 for SAFB, BCLAF1, ILF3, and DDX17. (**D**) Gene Ontology (GO) performed using the 4 HO-1 interactors with DNA-binding sites along with the target genes identified in ChIP-Atlas, displaying enriched KEGG pathways. The size of the points represents the number of observed genes for each category, and the color gradient represents the *p*-value. Statistical significance was set at *p* < 0.05.

To identify nuclear interactors of HO-1 in the context of PCa, we first performed a differential extraction of nuclear−cytoplasmic proteins from PC3 cells treated with or without hemin, a specific HO-1 inductor of expression and nuclear translocation [14]. Next, we co-immunoprecipitated HO-1 and subjected the output to proteomics analysis using LC-ESI-MS/MS to define its interactors, demonstrating a direct or indirect physical interaction between these proteins and HO-1 in the nucleus of PCa cells. This approach identified 44 proteins present in the nuclear fraction of PCa cells that were physically bound to HO-1, 11 of which were exclusively detected under hemin treatment (Figure 1A). Since HO-1 acquires nuclear localization upon hemin treatment and its function in this subcellular localization is not well defined, we focused on the 11 interactors (BCLAF1, SAFB, DDX17, ILF3, ILF2, CASP14, PDRX1, SLC25A5, CDC175, and BRIX1) found in the nucleus of hemin-treated cells and its interactions were validated for BCLAF1 and SAFB as shown in Supplementary Figure S1A,B. These interactors are involved in diverse biological functions, including chromatin organization and gene expression regulation [19,20], RNA stabilization [21,22], RNA metabolism regulation [23], ribosomal biogenesis [24], programmed cell death [25], centrosome control and microtubule assembly [26], redox sensing [27], and adenine nucleotide transport in mitochondria [28].

Then, we conducted an IPA [23] to explore the association between these interactors and HO-1, as well as their subcellular location. Consistent with the proteomics findings, IPA revealed that SAFB, BCLAF1, ILF3, ILF2, DDX17, and BRIX1 are present in the nucleus and constitute a network of direct and indirect interactions, with ILF3 linking HO-1 to this nuclear network (Figure 1B). PRDX1, GPATCH1, SLC25A5, CASP14, and CCDC175 have not previously been reported to localize to the nucleus or to interact with one another. Nevertheless, our findings identify these proteins as components of the HO-1 nuclear interactome, suggesting previously unrecognized associations that may provide novel insights into the non-canonical functions of HO-1.

### 2.2. Biological Characterization of HO-1 Nuclear Interactors

Considering that HO-1 regulates gene expression but lacks DNA-binding domains [16], we investigated the ability of HO-1 nuclear interactors to modulate gene expression as transcription factors or transcriptional co-regulators. The analysis using the ChIP-Atlas database revealed that BCLAF1, SAFB, ILF3, and DDX17 bind to DNA (Figure 1C(i,ii)), supporting their potential involvement in transcriptional regulation. To further characterize the functional implications of these interactors, we performed GO analysis to explore the KEGG database of biological pathways. The input gene list included HMOX1, the genes encoding the DNA-binding interactors (BCLAF1, SAFB, ILF3, and DDX17), and their target genes, resulting in a total of 799 genes analyzed (Supplementary Table S1). Unexpectedly, enrichment analysis revealed a significant overrepresentation of genes involved in neurodegenerative pathways, including Huntington’s disease, neurotrophin signaling, and choline metabolism in cancer (Figure 1D). Given the strong enrichment of neuronal-related programs, we next investigated the association between HMOX1, its nuclear interactors, and their target genes across distinct PCa subtypes, with a particular focus on NEPC.

### 2.3. HO-1 and Its Interactors Are Associated with NEPC

**Figure 2:**
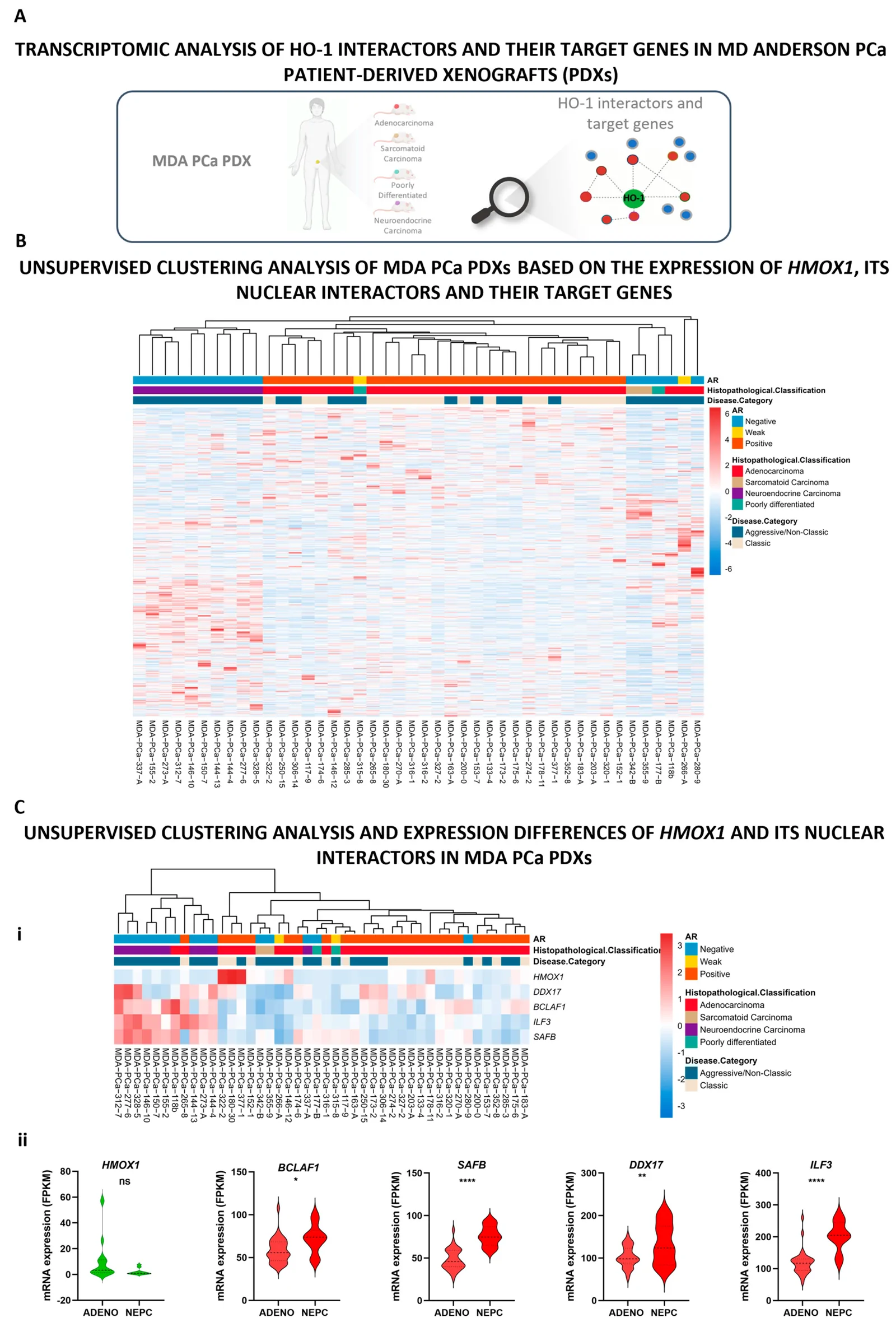
Transcriptomic analysis of the MDA PCa PDX series. (**A**) Schematic representation of the MDA PCa PDX series establishment and transcriptome analysis (n = 44), where HO-1 is represented as a green circle, its interactors in red and the target genes of the interactors in blue. (**B**) Heatmap depicting unsupervised clustering analysis of RNA-seq data from the 44 MDA PCa PDX series considering the expression of HO-1 nuclear interactors and its target genes (n = 799). Red, white, and blue represent greater, intermediate, and lower gene expression levels represented as z scores, respectively. Clinico-pathological variables corresponding to each patient are shown at the top of the heatmap and detailed on the right of the graph. (**C**): (**i**) Heatmap representing the mRNA expression of *HMOX1*, *BCLAF1*, *SAFB*, *DDX17*, and *ILF3*; (**ii**) violin plots comparing the expression levels (FPKM) of *HMOX1*, *SAFB*, *BCLAF1*, *DDX17*, and *ILF3* in adenocarcinoma (ADENO) and neuroendocrine prostate cancer (NEPC) samples. Statistical significance was set at *p* < 0.05, with * *p* < 0.05; ** *p* < 0.01; **** *p* < 0.0001, ns = not significant.

Using RNA-seq data from the clinically annotated, comprehensive MDA PCa PDX series [29], we assessed the expression levels of the abovementioned 799 genes (Figure 2A). Strikingly, the expression of these genes grouped PDXs according to their histopathological classification (adenocarcinoma, sarcomatoid, or poorly differentiated vs. neuroendocrine tumors), disease category (aggressive/non-classic vs. classic), and AR expression (negative, weak, or positive) in an unsupervised clustering analysis (Figure 2B).

Remarkably, when assessing only HMOX1, BCLAF1, SAFB, ILF3, and DDX17, most of the NEPC PDXs continued to cluster together in a single group with higher expression of the interactors and low HMOX1 expression, despite the substantial reduction in the number of genes included in the analysis (Figure 2C). These results were also observed in a PCA, which highlighted ILF3 as the gene which contributes the most to the variance between samples of different histopathological profiles followed by SAFB, BCLAF1, and DDX17 (Supplementary Figure S2).

Within the MDA PCa PDX collection, two models, MDA PCa 146-10 and MDA PCa 146-12, were derived from distinct histological regions of the same primary tumor, corresponding to NEPC and adenocarcinoma components, respectively (Supplementary Figure S3A). RNA-seq analysis revealed that PDX 146-12, established from the adenocarcinoma region, displayed higher HMOX1 expression together with lower expression of BCLAF1, SAFB, ILF3, and DDX17 compared with PDX 146-10, which originated from the neuroendocrine region of the tumor (Supplementary Figure S3B).

**Figure 3:**
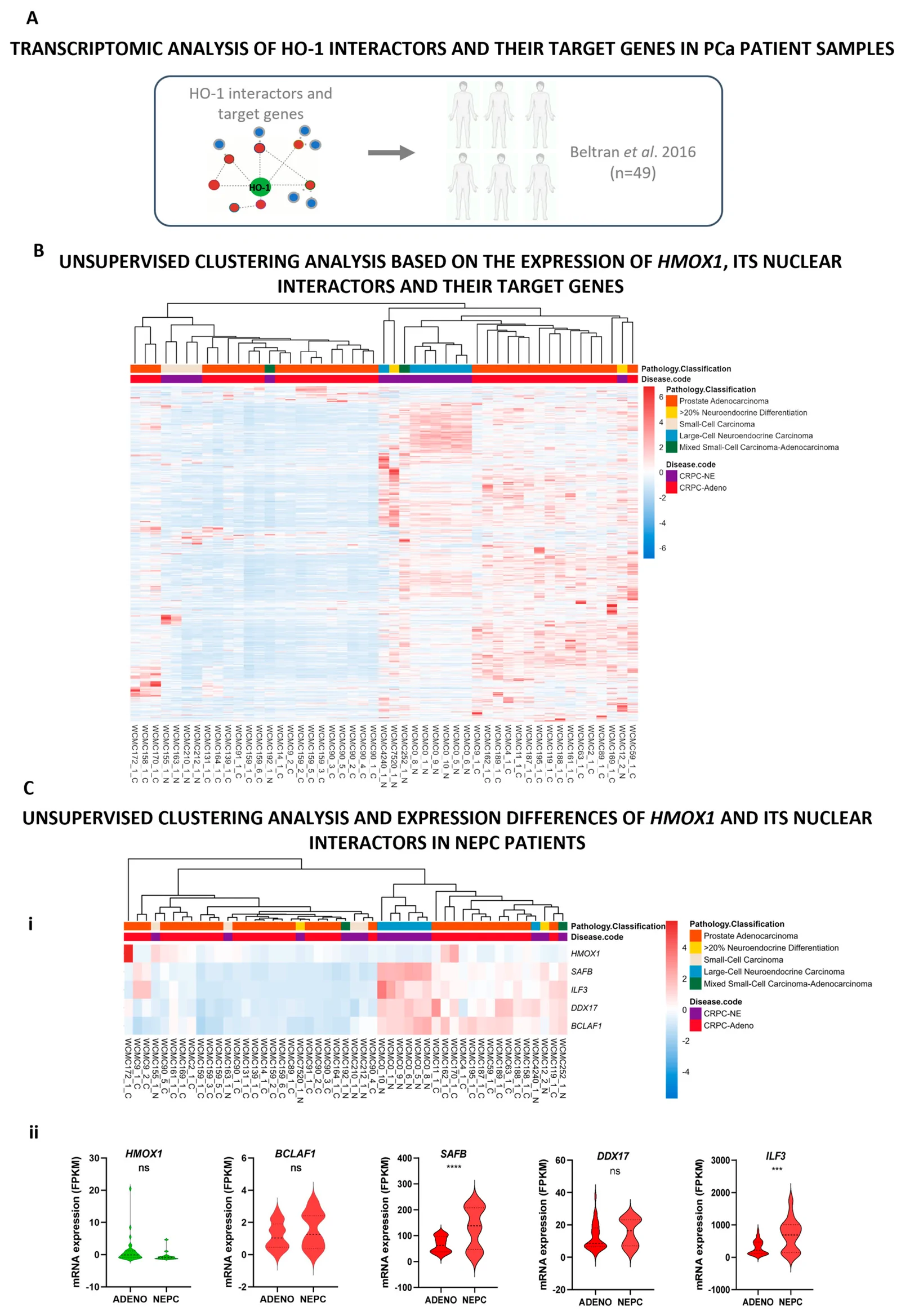
Transcriptomic analysis of patient samples. (**A**) Schematic workflow of RNA-seq data analysis of human patients in the dataset published by Beltran et al. (n = 49) [4], which comprises transcriptomic and clinical data from 15 neuroendocrine prostate cancer (CRPC-NE) tumors and 34 prostate adenocarcinomas (CRPC-Adeno) samples. (**B**) Heatmap showing an unsupervised clustering analysis of patient samples considering the gene expression of HO-1 nuclear interactors and its target genes (n = 799). Red, white, and blue represent greater, intermediate, and lower gene expression levels represented as z scores, respectively. Clinico-pathological variables corresponding to each patient are shown at the top of the heatmap and detailed on the right of the graph. (**C**): (**i**) Heatmap showing the expression (FPKM) of *HMOX1*, *SAFB*, *BCLAF1*, *DDX17*, and *ILF3*; (**ii**) violin plots showing the expression levels (FPKM) of *HMOX1*, *SAFB*, *BCLAF1*, *DDX17*, and *ILF3* in adenocarcinoma (ADENO) and neuroendocrine prostate cancer (NEPC) samples from the dataset published by Beltran et al. Statistical significance was set at *p* < 0.05; *** *p* < 0.001; **** *p* < 0.0001; ns = not significant.

To validate the association between *HMOX1*, its nuclear interactors, and the NE subtype, we analyzed the dataset published by Beltran et al. (n = 49) [4], which comprises transcriptomic and clinical data from 15 neuroendocrine prostate cancer (CRPC-NE) tumors and 34 prostate adenocarcinomas (CRPC-Adeno) samples (Figure 3A). Unsupervised clustering using either the 799-gene set or the 5-gene signature revealed a distinct cluster predominantly composed of CRPC-NE tumors, particularly large-cell NEPC samples (Figure 3B,C). Moreover, individual gene expression analyses demonstrated significant upregulation of *SAFB* and *ILF3* in NE samples compared with in adenocarcinoma samples, while *HMOX1*, *BCLAF1*, and *DDX17* showed no significant differences between groups (Figure 3C(ii)).

**Figure 4:**
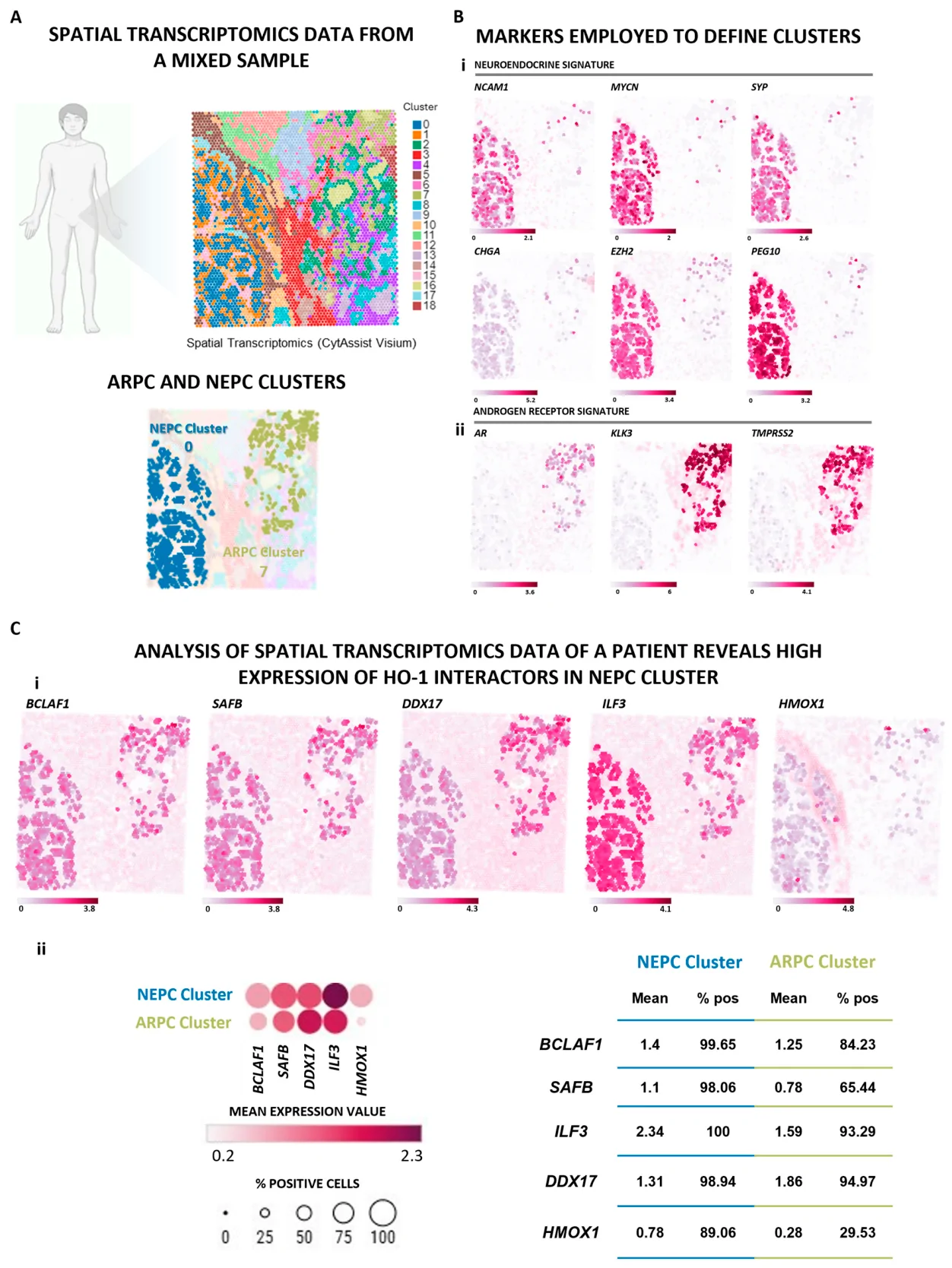
Assessment of the expression of HMOX1 interactors and its correlation with the NEPC phenotype using spatial transcriptomics data. (**A**) Schematic representation of the spatial gene expression analysis (CytAssist Visium) of a sample obtained from a 78-year-old patient with mixed carcinoma depicting de novo metastatic NEPC coexisting with AR pathway-positive adenocarcinoma (ARPC). The Gleason score was 4 + 4 [32]. Details of the clusters identified in the analyzed sample: cluster 0 (NEPC, blue) and cluster 7 (ARPC, green). (**B**): (**i**) Plots showing the neuroendocrine signature (*NCAM1*, *MYCN*, *SYP*, *CHGA*, *EZH2*, and *PEG10*) expressed in the tumor region classified as NEPC (cluster 0) and (**ii**) the androgen receptor signature (*AR*, *KLK3*, and *TMPRSS2*) expressed in the ARPC region (cluster 7). (**C**): (**i**) Spatial analysis of the expression of *BCLAF1*, *SAFB*, *ILF3*, *DDX17*, and *HMOX1*; (**ii**) dot-blot and table represent the mean expression value of *BCLAF1*, *SAFB*, *DDX17*, *ILF3*, and *HMOX1* and the percentage of positive cells (% pos) within each cluster.

To further characterize this association in a spatial context, we analyzed the expression patterns of *HMOX1* and its nuclear interactors using spatial transcriptomics data from the STOmics DB repository [30,31], derived from a patient harboring a mixed prostate cancer tumor composed of both NEPC and AR-positive prostate cancer (ARPC) regions (Figure 4A). To delineate and confirm the NEPC region within the tissue, we assessed the expression of characteristic NE markers (*NCAM1*, *MYCN*, *SYP*, *CHGA*, *EZH2*, and *PEG10*), which showed higher expression in cluster 0 (Figure 4B(i)) and a consistent downregulation in cluster 7 which was defined by high expression of *AR* and targets *KLK3* and *TMPRSS2* (Figure 4B(ii)) [32,33]. Regarding the spatial distribution of HO-1 nuclear interactors across the different areas of the tissue, *BCLAF1*, *SAFB*, and *ILF3* showed higher expression in cluster 0 (NEPC) compared to in cluster 7 (ARPC), whereas *DDX17* exhibited an opposite pattern (Figure 4C(i,ii)). Upon analyzing *HMOX1*, its expression was found to be higher in the NEPC region compared to in the ARPC region (Figure 4C(i,ii)). Watanabe et al. [32] mentioned that the NEPC region was surrounded by a highly fibrotic tissue area represented by cluster 5 (Figure 4A(i)), which presents elevated expression of fibrotic markers like *HMOX1* [32]. This tumor microenvironment might influence the expression of *HMOX1* detected in the NEPC region, which is higher than what we expected based on our previews results.

Together, these results highlight an association between HO-1 and NEPC, probably mediated by a non-canonical role exerted through its nuclear interactors.

### 2.4. HO-1 Induction Attenuates Molecular and Phenotypic NE Features Acquired upon Fsk-Induced Transdifferentiation in PCa Cells

**Figure 5:**
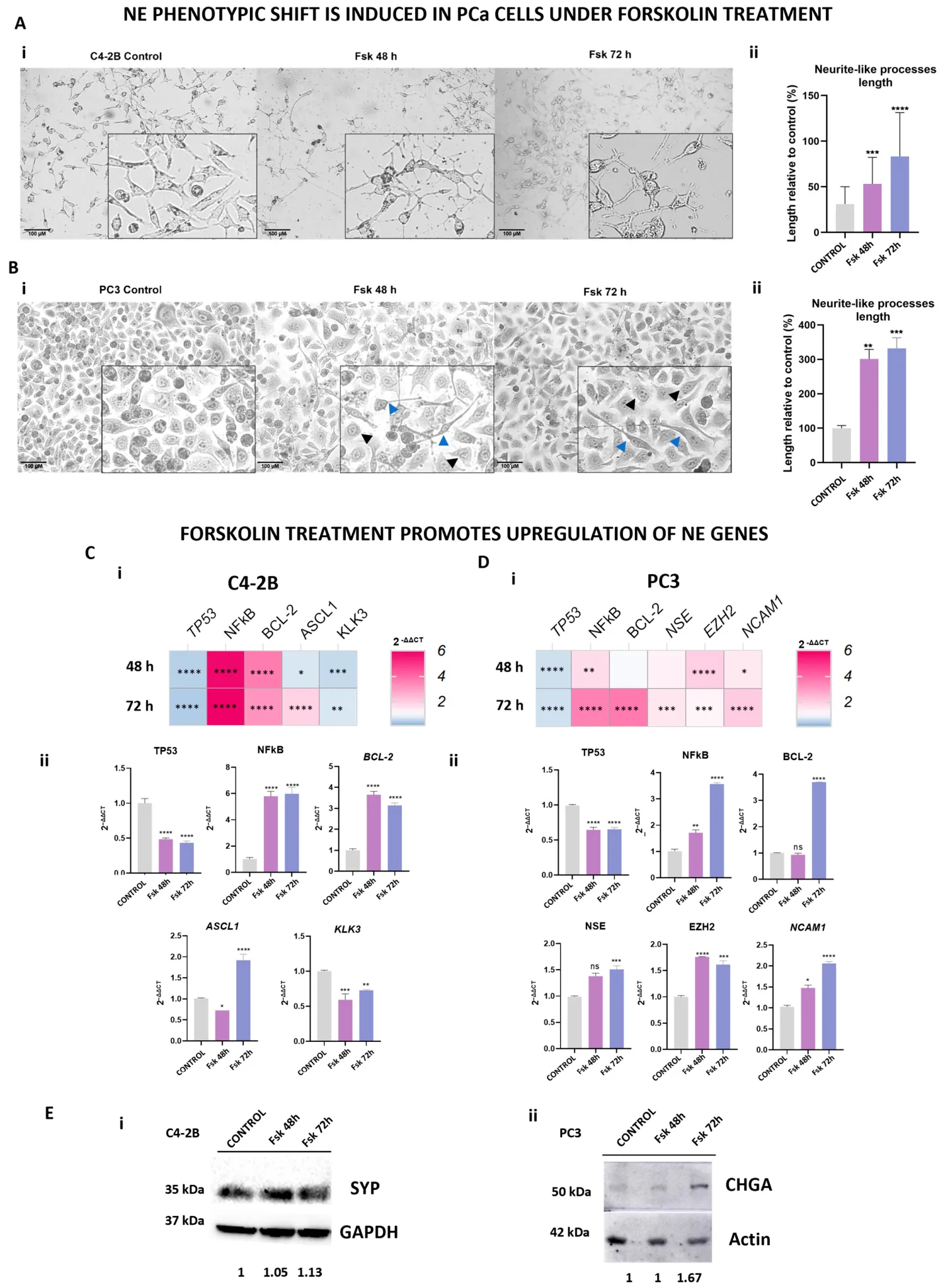
Transdifferentiation protocol established for PCa cells using Forskolin (Fsk). (**A**(**i**),**B**(**i**)) Representative images of C4-2B (**A**(**i**)) and PC3 (**B**(**i**)) cells for each treatment. Cells were treated with 10 µM Fsk for 48 h and 72 h. Micrographs were obtained using an inverted microscope equipped with a 10x air objective and analyzed with Fiji (version 1.54t, Bethesda, MD, USA) software. (**B**(**i**)) Two subpopulations are observed in response to Fsk treatment: one that retained the epithelial phenotype (black arrowheads) and another with a more elongated morphology featuring protrusions like neurites (blue arrowheads). (**A**(**ii**),**B**(**ii**)) Quantification of neurite-like processes length of C4-2B cells (**A**(**ii**)) and neurite-like processes length in PC3 cells (**B**(**ii**)). (**C**) Expression levels of *TP53*, *NFκB*, *BCL2*, *ASCL1*, and *KLK3* were measured by RT-qPCR in C4-2B cells treated with 10 µM Fsk for 48 h and 72 h and are depicted in a heatmap (**i**) and barplots (**ii**). (**D**) Expression levels of *TP53*, *NFκB*, *BCL-2*, *NSE*, *EZH2*, and *NCAM1* and were measured by RT-qPCR in PC3 cells after treatments and are depicted in a heatmap (**i**) and barplots (**ii**). *PPIA* was used as the reference gene for C4-2B cells and *GAPDH* for PC3 cells. Each condition is shown relative to its corresponding control. Results are presented as mean ± S.E.M. Statistical significance was set at *p* < 0.05; * *p* < 0.05; ** *p* < 0.01; *** *p* < 0.001; **** *p* < 0.0001; ns = not significant. (**E**) Western blot analysis of SYP in C4-2B cells (**i**) and CHGA in PC3 cells (**ii**). Cells were treated with 10 uM Fsk for 48 h and 72 h. GAPDH or actin were used as loading controls. Quantification values below each blot represent normalized expression relative to the untreated control.

Collectively, the whole transcriptomics, spatial transcriptomics, and bioinformatics analyses consistently associated the HO-1 nuclear interactome with NEPC features, suggesting a potential role for HO-1 in regulating lineage plasticity and NE transdifferentiation. To functionally investigate whether HO-1 modulation could directly impact the acquisition of NE characteristics in PCa cells, we next established an in vitro model of NE transdifferentiation based on cAMP pathway activation using forskolin (Fsk) [34]. C4-2B and PC3 cells were treated with Fsk (10 µM, 48 h, and 72 h), and morphology and molecular markers were analyzed (Figure 5).

Regarding the androgen-sensitive model, C4-2B Fsk-treated cells showed pronounced morphological changes compared with untreated controls, displaying a more elongated shape, featuring neurite-like processes (Figure 5A). In androgen-insensitive PC3 cells, two subpopulations were observed in response to Fsk treatment: one that retained the epithelial phenotype and another with a more elongated morphology featuring protrusions like neurites (Figure 5B).

These morphological changes were paired with changes in the expression of genes involved in NE transdifferentiation (Figure 5C,D). In both cell lines, we observed significant and sustained downregulation of *TP53*, a gene commonly altered in NEPC samples [35], and upregulation of *BCL2* and *NFκB* (Figure 5C,D). Downregulation of *KLK3* was detected in Fsk-treated C4-2B (Figure 5C).

With respect to NE markers, significant induction of *ASCL1* was detected in C4-2B cells (Figure 5C), and significant induction of *NSE*, *NCAM1*, and *EZH2* was observed in PC3 cells (Figure 5D) after 72 h Fsk treatment. The NE transdifferentiation was also assessed by measuring the canonical NE protein expression (SYP, CHGA) by Western blot (Figure 5E).

These results reveal molecular NE adaptations accompanying morphological alterations depending on the cell model upon Fsk treatment.

**Figure 6:**
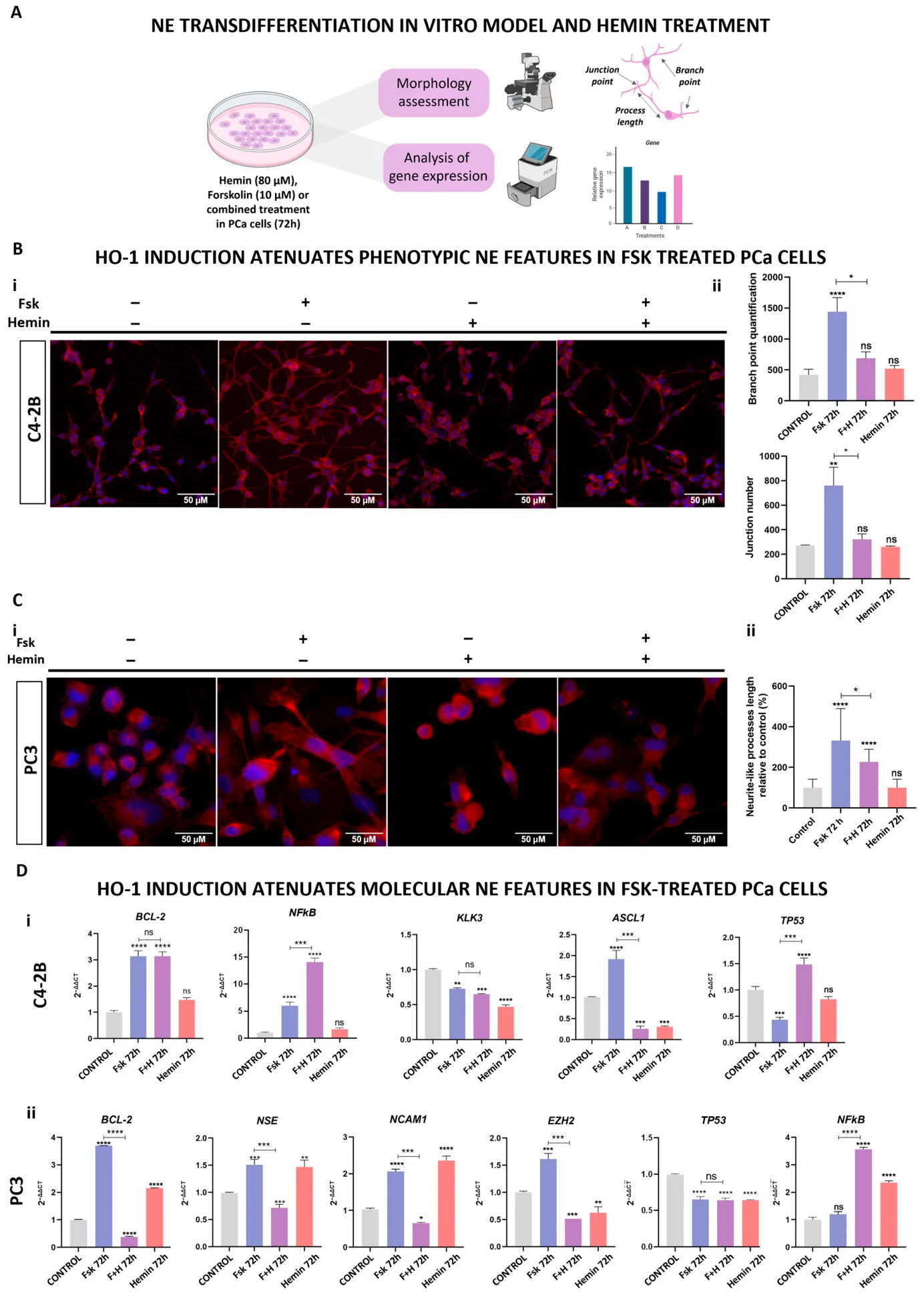
HO-1 pharmacological induction in transdifferentiated PCa cells. (**A**) Schematic representation of treatments employed and parameters measured in C4-2B and PC3 cells. (**B**(**i**),**C**(**i**)) Representative images of C4-2B (**B**(**i**)) and PC3 (**C**(**i**)) cells for each treatment. Cells were treated with 10 µM Fsk, 80 µM hemin, and co-treated with both drugs (F+H) for 72 h, and then were fixed and stained with TRITC–phalloidin and DAPI. Micrographs were obtained using an inverted epi-fluorescence microscope (Olympus IX71) equipped with a 10x air objective and analyzed with FiJi software. (**B**(**ii**),**C**(**ii**)) Quantification of branch points and junction number of C4-2B cells (**B**(**ii**)) and neurite-like processes length in PC3 cells (**C**(**ii**)) after 10 µM Fsk, 80 µM hemin, and co-treated with both drugs for 72 h. (**D**): (**i**) Expression levels of *BCL-2*, *KLK3*, *NFκB*, *ASCL1*, and *TP53* were measured by RT-qPCR in C4-2B and cells treated with 10 µM Fsk, 80 µM hemin, or the combination of both drugs for 72 h; (**ii**) expression levels of *BCL-2*, *NSE*, *NCAM1*, *EZH2*, *TP53*, and *NFκB* were measured by RT-qPCR in PC3 cells after treatments. *PPIA* was used as the reference gene for C4-2B cells and *GAPDH* was used for PC3 cells. Each condition is shown relative to its corresponding control. Results are presented as mean ± S.E.M. Statistical significance was set at *p* < 0.05; * *p* < 0.05; ** *p* < 0.01; *** *p* < 0.001; **** *p* < 0.0001*;* ns = not significant.

Having established this in vitro model of NE transdifferentiation, we next sought to determine the role of nuclear HO-1 in this process. Since hemin-induced HO-1 expression and nuclear translocation have been extensively characterized in PCa cells and linked to decreased proliferation as well as transcriptional programs regulating tumor morphology and cell adhesion [11,12,13,14,36], we co-treated cells with Fsk and hemin to evaluate whether HO-1 induction could modulate the acquisition of NE features (Figure 6A). In both cell lines, co-treatment with hemin significantly reduced the morphological features associated with NE phenotype induced by Fsk treatment (Figure 6B,C).

This impairment of NE morphology by HO-1 induction was also evidenced in the alteration of expression of transdifferentiation-induced molecular markers (*ASCL1*, *TP53*, and *NFκB* in C4-2B (Figure 6D(i)), and *NSE*, *NCAM1*, *EZH2*, *NFκB*, and *BCL2* in PC3 (Figure 6D(ii)).

Interestingly, Fsk-induced NE transdifferentiation in PC3 cells was accompanied by a significant increase in *BCLAF1*, *SAFB*, *ILF3*, and *DDX17* transcript levels, whereas *HMOX1* expression remained unchanged. Co-treatment with hemin markedly induced *HMOX1* expression and attenuated the Fsk-associated increase in the four HO-1 nuclear interactors (Supplementary Figure S4A).

Considering that, in our IPA analysis, ILF3 links HO-1 to the nuclear interactor network, and PCA of the MDA PCa PDX series identified ILF3 as the strongest contributor to the variance between samples of different histopathological profiles, we confirmed the nuclear localization of HO-1 and ILF3 during Fsk-induced transdifferentiation by confocal immunofluorescence analysis. Nuclear HO-1 fluorescence remained unchanged following Fsk treatment but markedly increased upon addition of hemin. In contrast, nuclear ILF3 fluorescence increased the following Fsk treatment and remained elevated in the co-treated condition, with no significant difference between Fsk and F+H treatments (Supplementary Figure S4B,C).

**Figure 7:**
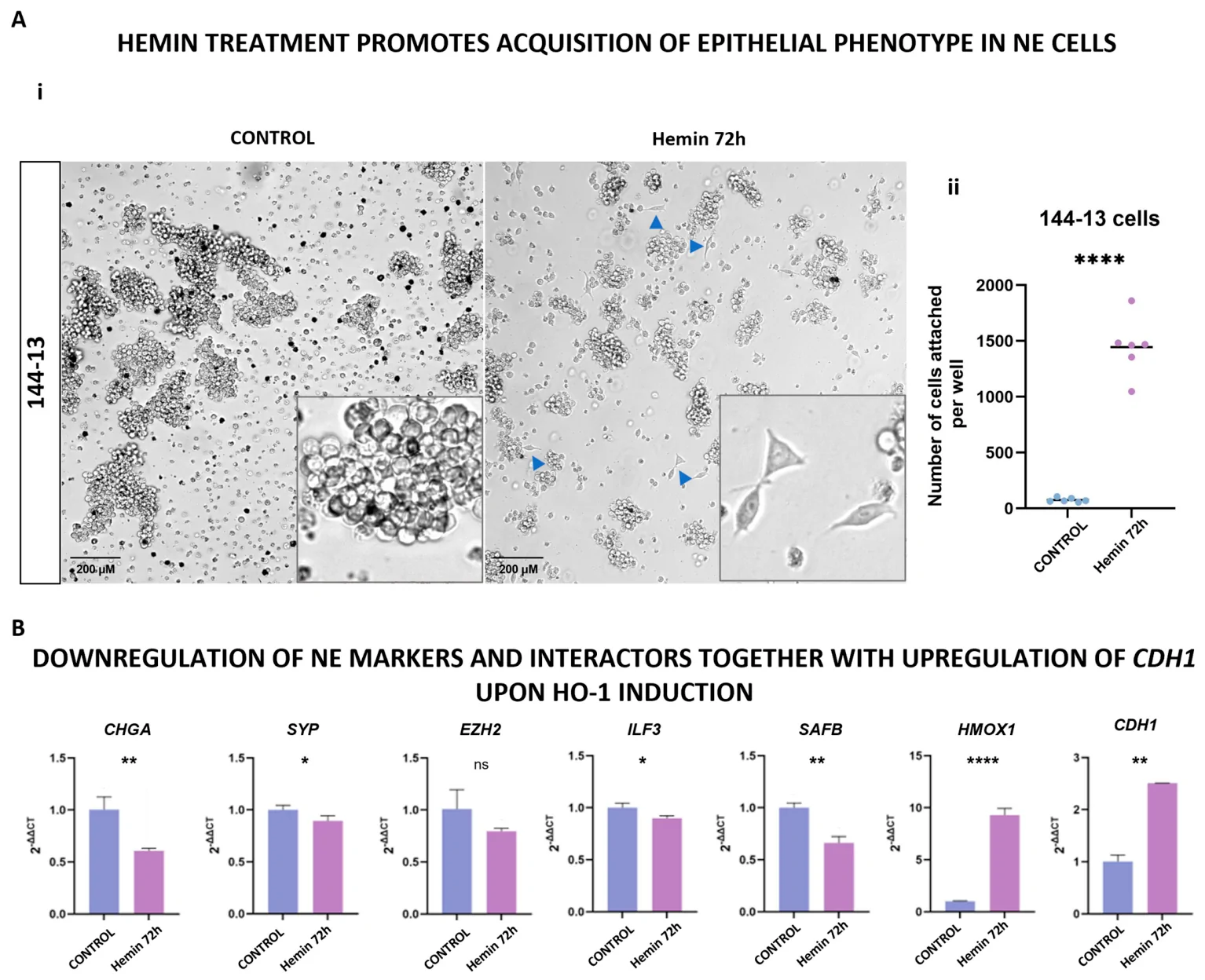
NEPC cells show phenotypic changes under hemin treatment. (**A**): (**i**) Representative bright-field micrographs of MDA PCa 144-13 cells for control and treatment conditions (hemin 80 μM; 72 h). Insets show magnified views of cell morphology. Images were acquired using an inverted microscope equipped with a 10× objective. Arrowheads point attached cells; (**ii**) quantification of the number of attached cells per well in control and hemin-treated conditions. (**B**) Expression levels of *CHGA*, *SYP*, *EZH2*, *ILF3*, *SAFB*, *HMOX1*, and *CDH1* were measured by RT-qPCR in MDA PCa 144-13 cells after treatments. *PPIA* was used as the reference gene. Each condition is shown relative to its corresponding control. Results are presented as mean ± S.E.M. Statistical significance was set at *p* < 0.05; * *p* < 0.05; ** *p* < 0.01; **** *p* < 0.0001*;* ns = not significant.

To further evaluate the effect of HO-1 induction in an established neuroendocrine context, we employed MDA PCa 144-13 cells, derived from the corresponding neuroendocrine MDA PCa PDX. Hemin treatment (80 μM, 72 h) induced a marked phenotypic shift, with cells transitioning from their characteristic rounded, cluster-forming morphology toward a more spread and adherent epithelial-like appearance (Figure 7A(i)).

Quantification of the attachment assay demonstrated a significant increase in the number of adherent cells following hemin treatment compared with control cells (Figure 7A(ii)). Consistent with this phenotypic change, RT-qPCR analysis showed significant downregulation of the neuroendocrine markers *CHGA* and *SYP*, whereas *EZH2* remained unchanged. Expression of the HO-1 nuclear interactors *ILF3* and *SAFB* was also significantly reduced. As expected, *HMOX1* was strongly induced by hemin treatment. Importantly, expression of the epithelial marker *CDH1* significantly increased, consistent with the enhanced attachment phenotype and supporting an HO-1 associated shift towards a more epithelial-like phenotype in MDA PCa 144-13 cells. Together, these results indicate that HO-1 induction attenuates established neuroendocrine features and promotes the acquisition of epithelial-like characteristics in MDA PCa 144-13 cells.

## 3. Discussion

The findings presented in this work demonstrate, for the first time, that HO-1 and its nuclear interactors play an active role in regulating tumor plasticity in PCa. Through a comprehensive integrative approach combining proteomic and transcriptomic analyses, preclinical models, patient-derived data, and in vitro transdifferentiation assays, we identified a set of nuclear interactors of HO-1 (BCLAF1, SAFB, ILF3, and DDX17) capable of modulating gene expression and associated with the aggressive clinical phenotype, NEPC.

The nuclear interactome of HO-1 identified in this work represents the first experimental map describing its nuclear associations in PCa cells. Many of these interactors are involved in chromatin organization, RNA processing, and transcriptional regulation, revealing a broad functional spectrum that extends far beyond the canonical cytoplasmic functions of HO-1 [20,23]. This molecular interaction network, validated by co-immunoprecipitation and bioinformatics analyses, demonstrates that HO-1 integrates into transcriptional complexes that control the expression of genes associated with differentiation, apoptosis, and structural remodeling, thereby supporting a role for HO-1 as a nuclear co-regulator. Coordinated expression of *HMOX1* and its interactors distinguished NEPC tumors in PDX models and clinical cohorts, redefining the role of HO-1 within the molecular profile of advanced PCa.

These results align with a growing body of evidence attributing non-canonical functions to HO-1, including gene regulation, DNA damage response, and epigenetic reprogramming [37,38]. Several authors have shown that nuclear translocation of HO-1 can modulate the expression of genes involved in cell adhesion, proliferation, and inflammatory responses [11,39]. However, until now, no link has been documented between this nuclear localization and lineage plasticity in PCa. These findings are particularly relevant in the current scientific landscape, which recognizes HO-1 as a dual-acting enzyme in cancer, capable of exerting either protective or pro-tumorigenic effects depending on the cellular context, the magnitude of oxidative stress, and its subcellular localization [38,40].

Pharmacological induction of HO-1 partially reversed NE transdifferentiation in two different PCa cell lines (PC3 and C42B) at both molecular and phenotypic levels. Further, assays performed in a well-established NEPC cell model (MDA PCa 144-13) showed that HO-1 induction was associated with a coordinated reduction in NE markers together with increased epithelial features. These results highlight the role of HO-1 as a negative regulator of the NE program. These observations are consistent with the CREB/STAT3 pathway involvement in NE reprogramming and support a model in which nuclear HO-1 interferes with this cascade, favoring luminal differentiation [13]. Collectively, these results provide a novel and multidimensional perspective on the role of HO-1, positioning it not merely as a cellular stress sensor but as a nuclear modulator with direct impact on PCa progression, intratumoral heterogeneity, and lineage differentiation. In this study, hemin treatment on transdifferentiated PCa cells diminished the morphological and molecular alterations promoted by Fsk, clearly associated with NE phenotypes. These findings extend previous evidence reported by our group, demonstrating that *HMOX1* overexpression reduces proliferation, migration, invasion, and tumor vascularization [11,12,14]. Through confocal immunofluorescence analysis, we were able to confirm the nuclear localization of HO-1 during Fsk-induced transdifferentiation in PCa cells. In line with the study by Ben-Eltriki et al. [40], our findings indicate that, under the experimental conditions employed, HO-1 limits NE transdifferentiation when its nuclear localization is induced. From a translational perspective, these findings suggest that controlled HO-1 induction could represent a strategy to prevent or reverse NE conversion, a major mechanism of resistance to next-generation anti-androgen therapies.

Analyses in PDX models and the Beltran et al. cohort revealed that low *HMOX1* combined with high expression of its nuclear partners strongly associates with the NEPC phenotype, suggesting that HO-1 acts as a transcriptional “buffer” that stabilizes epithelial identity and restrains NE reprogramming. Validation in PDX models and the dataset published by Beltran et al. [4] confirmed the clinical relevance of the *HMOX1*:*BCLAF1*:*SAFB*:*ILF3*:*DDX17* print. The five-gene signature stratified NEPC PDXs into a distinct cluster and reproduced the same pattern in human samples, with pre-clinical models showing that HO-1 loss correlates with histological transition to NE regions. The spatial transcriptomics analysis provided an additional layer of resolution by showing that *BCLAF1*, *SAFB*, and *ILF3* were enriched within NEPC regions of a mixed-histology tumor. However, *HMOX1* expression in this sample did not follow the inverse pattern observed in bulk PDX and clinical datasets. Rather than invalidating the signature, this finding underscores that *HMOX1* is a stress-responsive gene expressed not only by tumor cells but also by stromal, inflammatory, and fibrotic compartments. In the analyzed spatial dataset, the NEPC area was adjacent to a fibrotic region, which may contribute to the elevated *HMOX1* signal detected in NEPC-associated spots. Thus, spatial transcriptomics suggests that the HO-1 nuclear interactors are topographically linked to NEPC, whereas *HMOX1* expression requires compartment-aware interpretation.

In summary, this study establishes HO-1 as a nuclear regulator of tumor plasticity in PCa. By interacting with transcriptional co-factors, HO-1 modulates lineage identity and limits NE transdifferentiation. These results integrate and expand current knowledge on the functions of HO-1 in PCa, proposing a model in which the nuclear HO-1 interactome arises as a biologically relevant axis in NEPC and provides a framework for improving molecular stratification of aggressive PCa.

## 4. Materials and Methods

### 4.1. Cell Lines

Human PC3 (an androgen-insensitive cell line derived from a bone metastasis) and C4-2B (an androgen-sensitive cell line derived from the LNCaP PCa cell line) cell lines were obtained from the American Type Culture Collection (ATCC, Manassas, VA, USA). MDA PCa 144-13 cells (a neuroendocrine cell line that grew) derived from the MDA PCa patient-derived xenograft (PDX) were developed in the Prostate Cancer Patient-Derived Xenograft Program at The University of Texas MD Anderson Cancer Center [29]. These cell lines were routinely cultured using RPMI 1640 (Invitrogen, Grand Island, NY, USA) supplemented with 10% fetal bovine serum (FBS) (Internegocios, Mercedes, Buenos Aires, Argentina). Cultures were maintained at 37 °C in a humidified incubator with 5% CO_2_.

### 4.2. Cell Culture Treatments

For treatments, cells were incubated for 24 h and then exposed to vehicle, forskolin (Fsk) (10 µM), hemin (80 μM), or Fsk (10 µM) and hemin (80 μM) (F+H).

Hemin, an analog of the heme group and specific HO-1 inducer and activator [41], was obtained from Sigma-Aldrich (Glasgow, UK). A stock solution was prepared at a final concentration of 55 mM in PBS and 0.1 M NaOH. The solution was sterilized by filtration through 0.2 µm filters, aliquoted and stored at −20 °C until use.

Forskolin (Fsk) (Sigma-Aldrich, St. Louis, MO, USA) is a diterpene that activates adenylate cyclase and stimulates the production of intracellular cyclic adenosine 3′,5′-monophosphate (cAMP) [34]. A stock solution was prepared at a final concentration of 10 mM in dimethyl sulfoxide (DMSO). The solution was sterilized by filtration through 0.2 µm filters, aliquoted and stored at −20 °C until use. For cell-culture treatments, the stock solution was diluted to 10 µM in RPMI supplemented with 10% (*v*/*v*) FBS.

For proteomics analysis, cells were treated with hemin (80 μM) for 24 h. For the NE transdifferentiation of PCa cells, we adapted the protocol described by Zhang et al. [42]. Cells were treated with Fsk (10 µM) for different times (48 h, 72 h). For the co-treatment in the transdifferentiation assay, we adapted the hemin treatment time to align with the Fsk-induced transdifferentiation protocol. In these experiments, cells were exposed to Fsk (10 µM) and hemin (80 µM) for 72 h. In all cases, control cells were treated for the same time with the respective vehicle.

For attachment assays, we plated 2.5 × 10^5^ MDA PCa 144-13 cells growing in suspension in a 12 multiwell plate. Wells were coated with Human FNC Coating Mix (AthenaES, Arbutus, MD, USA) to provide structural anchorage and an artificial microenvironment to promote the attachment. Cells were treated with hemin (80 μM) for 72 h. We counted the number of cells attached across six wells per condition.

### 4.3. Co-Immunoprecipitation (Co-IP) Assays

We established two experimental conditions, control cells and hemin-treated cells. After treatment, we performed a differential protein extraction resulting in cytoplasm and nuclear protein extracts. Protein quantification was performed using the BCA assay (Sigma Aldrich, Gillingham, UK; 98% BCA, 2% CuSO_4_). For each immunoprecipitation reaction, 500 µg of the nuclear protein extracts of control and hemin-treated cells was combined with 10 µg of rabbit anti-human HO 1 antibody in a final volume of 500 µL RIPA buffer (150 µM NaCl, 20 µM EDTA, 1% (*v*/*v*) sodium deoxycholate, 0.1% (*v*/*v*) SDS, 1% Triton X-100, and Tris (pH 7.4)). As a specificity control, parallel samples were incubated with 10 µg of nonspecific rabbit anti-human IgG. All samples were supplemented with MPI protease inhibitors (Sigma Aldrich, Gillingham, UK) and incubated overnight at 4 °C with orbital agitation.

Protein A/G PLUS-Agarose beads (Santa Cruz Biotechnology, Santa Cruz, CA, USA) were pre-washed, and 20 µL of beads was added to each sample, followed by a second overnight incubation at 4 °C with orbital shaking. Antibody/protein/bead complexes were washed three times with 500 µL of RIPA buffer and centrifuged at 2500 rpm for 1 min at 4 °C. The protein/bead complexes were resuspended in a final volume of 40 µL of RIPA buffer and prepared for mass spectrometry analysis (MS/MS).

To validate the results obtained by MS/MS, a co-immunoprecipitation was performed using 500 µg of proteins from hemin-treated PC3 cell lysates. Briefly, the beads were vortexed, washed on a magnet, and incubated with 10 µg of either specific antibodies (anti-SAFB or anti-BCLAF1) or non-specific IgG as the control. Antibody/bead complexes were then incubated with the protein lysates for antigen capture. Afterward, the beads were added to the antibody-containing lysate and incubated again under orbital rotation. The beads were magnetized, the unbound fraction was removed, and the complexes were washed three times with washing buffer at 4 °C. Finally, protein complexes were eluted with elution buffer, neutralized with Tris-HCl (pH 8.0) and analyzed by Western blot.

### 4.4. Mass Spectrometry Analysis

Peptides were analyzed by nano-LC-ESI-MS/MS using a Q Exactive mass spectrometer coupled to an EASY-nLC 1000 nano-HPLC system (Thermo Scientific). For LC-ESI-MS/MS analysis, approximately 1 µg of peptides was loaded onto the column and eluted over 120 min using an appropriate reverse-phase column (C18, 2 µm, 100 Å, 50 µm × 150 mm, Easy-Spray PepMap RSLC, P/N ES801), allowing high-resolution separation of protein complexes. The nano-column flow rate was set to 300 nL min^−1^, and the solvent gradient ranged from 7% solvent A (0.1% formic acid in water) for 5 min to 35% solvent B (0.1% formic acid in acetonitrile) over 120 min. The injection volume was 2 µL.

The mass spectrometer is equipped with a high-energy collision dissociation (HCD) cell for fragmentation and an Orbitrap analyzer (Q-Exactive, Thermo Scientific, Bremen, Germany). A spray voltage of 3.5 kV was applied for electrospray ionization (Thermo Scientific EASY-SPRAY). Data acquisition was performed using XCalibur 3.0.63 (Thermo Scientific) under conditions that enabled peptide identification concurrently with chromatographic separation. A data-dependent acquisition method was employed: full-scan MS spectra were acquired in the Orbitrap analyzer over a mass range of 400–1800 *m*/*z* at a resolution of 70,000 at 400 *m*/*z*. The twelve most intense ions in each cycle were sequentially isolated, fragmented by HCD, and analyzed in the Orbitrap analyzer. Singly charged peptides and ions with unassigned charge states were excluded from MS/MS fragmentation. The MS data were analyzed using Proteome Discoverer software (version 2.1.1.21; Thermo Scientific), employing the Sequest search engine, against *Homo sapiens* protein sequences from the Uniprot database. Search parameters included trypsin digestion with 1 miscleavage, fixed carbamidomethylation of cysteines and variable oxidation of methionines as post-translational modifications. The search allowed a parent ion tolerance of 10 ppm and a fragment mass tolerance of 0.05 Da and peptides were identified with a false discovery rate of less than 1%, calculated using a concatenated decoy database.

### 4.5. Western Blot

Immunoblots were carried out as described by Anselmino et al. [43] using the following primary antibodies: anti-β-actin (cat. #22952; Proteintech; Rosemont, IL, USA; rabbit; 1:1000), anti-GAPDH (cat. #3873; Cell Signaling; Danvers, MA, USA; mouse; 1:1000), anti-CHGA (cat. #32127; Abcam; Waltham, MA, USA; rabbit; 1:1000), anti-SYP (cat. #32127; Abcam; Waltham, MA, USA; rabbit; 1:1000), anti-HO-1 (cat. #13248; Abcam; Waltham, MA, USA; mouse; 1:1000), anti-BCLAF1 (cat. #bs-7583R-PE; Bioss; Woburn, MA, USA; rabbit; 1:1000), and anti-SAFB (cat. #bs-17259R; Bioss; Woburn, MA, USA; rabbit 1:1000). Secondary antibodies included HRP-conjugated anti-rabbit IgG (cat. #7074S; Cell Signaling; Danvers, MA, USA; 1:5000) and anti-mouse IgG (cat. #7076S; Cell Signaling; Danvers, MA, USA; 1:5000). Densitometry was performed using Fiji (Bethesda, MD, USA) software and band intensities were normalized to the loading control.

### 4.6. Ingenuity Pathway Analysis

Ingenuity Pathway Analysis (IPA, QIAGEN Inc., Germantown, MA, USA) was used to study the interactions between HO-1 and its nuclear interactors and with other molecules [44].

### 4.7. ChIP Atlas

To identify the target genes of proteins with a potential transcription-factor function, the target genes tool in the ChIP-Atlas webtool (https://chip-atlas.org/target_genes, accessed on 12 October 2023) was used. This tool integrates public ChIP-seq data to predict-binding sites in target genes based on enriched binding regions. The search was performed for the 11 nuclear interactors of HO-1, and parameters such as species and the distance from the transcription start site were defined. A maximum distance of 5 Kb from the transcription start site was selected (typically 1–10 Kb). The tool processes ChIP-seq datasets, identifying genes with binding peaks near the promoter, and generates a list of target genes ranked according to a binding score for each gene. This binding score quantifies the transcription factor’s binding strength based on the intensity and position of the ChIP-seq peaks.

### 4.8. Gene Ontology (GO)

GO was performed using the R packages clusterProfiler (version 4.18.4) [45] and enrichplot (version 1.30.5) [46]. The KEGG pathway collection was used to identify enriched pathways, and pathways with *p* < 0.05 were considered significant.

### 4.9. Transcriptomics Analysis of MDA PCa PDXs

To determine the association of HO-1 and its interactors with the different PCa subtypes we explored the PDXs collection developed at the Laboratory of Dr. Navone within the “Prostate Cancer Patient Derived Xenograft Program” at MD Anderson Cancer Center (MDA PCa PDXs) and the David H. Koch Center for Applied Research of Genitourinary Cancers. The MDA PCa PDXs include detailed clinicopathological data from the original patients, along with transcriptomic and genomic profiles of the corresponding PDXs [29].

PDXs 146-10 and 146-12 were derived from different regions of a single mixed adeno-NEPC primary tumor and subsequently established as pure NEPC (146-10) and adenocarcinoma (146-12) PDXs. The NEPC PDX 146-10 does not express *AR* or its downstream target *ZBTB16*.

### 4.10. Human NEPC Samples

Transcriptomics data from the Neuroendocrine Prostate Cancer (Multi-Institute, Nat Med 2016) dataset published by Beltran et al. [4] in cBioPortal were explored [47,48,49]. This dataset presents human NEPC samples and clinical data from patients, including an extensive histopathological classification of the samples.

### 4.11. Unsupervised Clustering and Principal Component Analysis (PCA)

Unsupervised clustering analysis including the expression data of the HO-1 nuclear interactors and target genes were performed using the pheatmap (version 1.0.12) [50] package. PCA and variable contributions were calculated using the factoextra (version 1.0.7) [51] package in R.

### 4.12. Spatial Transcriptomics Data Analysis

The spatial transcriptomic data from Watanabe et al. [32] were explored using STOmicsDB (https://db.cngb.org/stomics/, accessed on 20 January 2025) [31], a comprehensive database for spatial transcriptomic data sharing, analysis, and visualization. The platform’s visualization interface was used to inspect spatial gene-expression patterns and to compare transcriptomic features between NEPC clusters and ARPC clusters [30].

### 4.13. Cellular Staining and Morphological Evaluation

Morphological changes upon drug treatments were assessed after cells were stained with phalloidin-TRITC to increase contrast between cellular features and the background.

Briefly, 60,000 PCa cells were cultured on coverslips placed in 12-well plates and the respective treatments were carried out. Afterwards, cells were washed three times with PBS and then fixed with 500 µL of 4% paraformaldehyde (PFA) for 20 min at room temperature. After fixation, cells were incubated in a permeabilization solution (PBS with 0.05% *v*/*v* Triton X-100) for 15 min and washed twice with PBS before phalloidin-TRITC staining (Life Technologies (a brand of Thermo Fisher Scientific Inc.), Eugene, OR, USA) using a working dilution of 1:100. Micrographs were obtained using an inverted epi-fluorescence microscope (Olympus IX71) equipped with a 10x air objective and analyzed using the Skeletonize plugin in Fiji (Bethesda, MD, USA), which provided information about branch points, branch junctions, and neurite-like processes length.

### 4.14. Immunofluorescence (IF)

HO-1 nuclear localization was assessed by immunofluorescence using the protocol described in Lage-Vickers et al. [15]. We used anti-HO-1 (cat. #ab13243; Abcam; Waltham, MA, USA; 1:150) and fluorescent secondary antibody *Cy2* goat anti-rabbit IgG (cat. # ab6940; Abcam; Waltham, MA, USA; 1:250). Cells were counterstained with DAPI and images were acquired using a Zeiss LSM980 confocal microscope equipped with a 63× oil-immersion objective (NA 1.4; Zeiss, Oberkochen, Germany). Micrographs were analyzed with Fiji (Bethesda, MD, USA) software.

### 4.15. RNA Isolation, cDNA Synthesis, and RT-qPCR

Total RNA was isolated with Quick-Zol (Kalium technologies, Buenos Aires, Argentina) according to the manufacturer’s protocol. cDNAs were synthesized with the RevertAid Premium First Strand cDNA Synthesis Kit (Thermo Scientific, Waltham, MA, USA) and used for real-time PCR amplification with Taq DNA Polymerase (Invitrogen, Waltham, MA, USA).

*PPIA* and *GAPDH* were used as internal reference gene for PC3 and C4-2B cells, respectively. Data obtained were analyzed using the method of 2^−ΔΔCT^ [52]. Primers sequences for each gene are shown in Table 1.

### 4.16. Statistical Analysis

All bioinformatics analyses were performed using R programming language through RStudio platform (version 2024.04.1, PBC, Boston, MA, USA). For graphics, the packages ggplot2 (version 3.5.1) [53], ggpubr (version 0.6.0) [54], and RColorBrewer (version 1.1-13) [55] were used. Bar, violin, and dot plots were created using GraphPad Prism (version 8.4.2, La Jolla, CA, USA). For morphological changes and gene expression analysis, ANOVA followed by Tukey post-hoc tests was performed to assess statistical significance with a threshold of *p* < 0.05 (*), *p* < 0.01 (**), *p* < 0.001 (***), and *p* < 0.0001 (****).

## 5. Conclusions

We defined the nuclear interactors of HO-1 by a proteomics approach and linked the *HMOX1*:*BCLAF1*:*SAFB*:*ILF3*:*DDX17* print with the NEPC phenotype in PDXs and PCa patient samples. We reported here, for the first time, the impairment of NE transdifferentiation exerted by HO-1 in PCa cells. This work underscores the clinical and biological significance of the HO-1 nuclear interactome in PCa phenotype plasticity.

In this study, we defined the nuclear HO-1 interactome in PCa cells through an integrative proteomic and bioinformatic approach and identified a nuclear HO-1-associated signature composed of HMOX1, BCLAF1, SAFB, ILF3, and DDX17 linked to the NEPC phenotype across PDX models and patient-derived samples. Functionally, we demonstrate for the first time that HO-1 induction attenuates neuroendocrine transdifferentiation and the acquisition of NEPC-associated molecular and phenotypic features in PCa cells. Altogether, our findings position the nuclear HO-1 interactome as a previously unrecognized regulator of prostate cancer lineage plasticity and provide a framework for improving the molecular characterization of aggressive neuroendocrine disease states.

### Limitations

Although our data support the anti-tumoral role of HO-1 in PCa, the dual function reported in cancer highlights the importance of tissue and context-specific validation before broader therapeutic inferences can be made. Despite the nuclear localization of HO-1 and its antitumoral role, we cannot discard the contribution of this enzyme derived from its canonical cytoplasmic activity. Further, despite the consistent association observed across patient-derived datasets and in vitro models, additional in vivo studies specifically addressing therapy-induced NEPC progression will be important to determine the biological and therapeutic relevance of HO-1 modulation during lineage transition. Finally, the spatial transcriptomics analysis was based on a single mixed-histology tumor; therefore, although it provides valuable topographic support for the enrichment of HO-1 nuclear interactors in NEPC regions, additional spatially resolved cohorts will be required to determine whether *HMOX1* expression reflects tumor-intrinsic regulation or microenvironmental/stromal contributions.

## Figures and Tables

**Table 1:** Primer sequences used to measure gene expression by RT-qPCR.

Gene	Primers	Annealing Temp
*ASCL1*	Fw: CGGTCTCATCCTACTCGTCG	59
	Rv: GTTGTGCGATCACCCTGCTT	
*BCL-2*	Fw: CCTGTGGATGACTGAGTA	60
	Rv: GAGACAGCCAGGAGAAATCA	
*EZH2*	Fw: CGAAGCTACTCCGAGTTCCC	59
	Rv: GCACTCAGACAAAGGACGGA	
*GAPDH*	Fw: CAGTCAGCCGCATCTTCTTTTG	60
	Rv: ACCAGAGTTAAAAGCAGCCCT	
*HMOX1*	Fw: ACTGCGTTCCTGCTCAACAT	60
	Rv: GGGGCAGAATCTTGCACTTT	
*KLK3*	Fw: TGAACCAGAGGAGTTCTTGAC	56
	Rv: CCCAGAATCACCCGAGCAG	
*NCAM1*	Fw: AGTCCAAGGGGAACCCAGT	60
	Rv: CTCAGCATTCCAGTCCAGGG	
*NFκB*	Fw: GCACCCTGACCTTGCCTATT	61
	Rv: GCTCTTTTTCCCGATCTCCCA	
*NSE*	Fw: TGCACAGGCCAGATCAAGAC	59
	Rv: CCAGGCAAGCAGAGGAATCA	
*PPIA*	Fw: GGTATAAAAGGGGCGGGAGG	60
	Rv: CTGCAAACAGCTCAAAGGAGAC	
*TP53*	Fw: TCCCCTGCCCTCAACAAGATG	55
	Rv: GAGAGGAGCTGGTGTTGTTGG	
*SYP*	Fw: CGGACATGGACGTGGTGAAT Rv: AGATGGCGAAGACCCATTGC	60
*CHGA*	Fw: CAAGACCTCGCTCTCCAA Rv: CTCTTCCACCGCCTCTTTCA	58
*ILF3*	Fw: GTAAAACAGCAGGGGCCGAT Rv: TCCATCCACTTCGACCTCCA	60
*SAFB*	Fw: AAGAGGTCTAGCAAAGGGCG Rv: CGACACAATCTGCAACGCTT	60
*CDH1*	Fw:TACCCTGGTGGTTCAAGCTG Rv: ACCTGACCCTTGTACGTGGT	62

## Data Availability

The original contributions presented in this study are included in the article/Supplementary Materials. Further inquiries can be directed to the corresponding authors.

## Supplementary Materials

The following supporting information can be downloaded at: https://www.mdpi.com/article/10.3390/ijms27177635/s1.

## References

[1] Bray F., Laversanne M., Sung H., Ferlay J., Siegel R.L., Soerjomataram I., Jemal A. (2024). Global Cancer Statistics 2022: GLOBOCAN Estimates of Incidence and Mortality Worldwide for 36 Cancers in 185 Countries. CA Cancer J. Clin..

[2] Logothetis C.J., Gallick G.E., Maity S.N., Kim J., Aparicio A., Efstathiou E., Lin S.H. (2013). Molecular Classification of Prostate Cancer Progression: Foundation for Marker-Driven Treatment of Prostate Cancer. Cancer Discov..

[3] Aggarwal R., Huang J., Alumkal J.J., Zhang L., Feng F.Y., Thomas G.V., Weinstein A.S., Friedl V., Zhang C., Witte O.N. (2018). Clinical and Genomic Characterization of Treatment-Emergent Small-Cell Neuroendocrine Prostate Cancer: A Multi-Institutional Prospective Study. J. Clin. Oncol..

[4] Beltran H., Prandi D., Mosquera J.M., Benelli M., Puca L., Cyrta J., Marotz C., Giannopoulou E., Chakravarthi B.V.S.K., Varambally S. (2016). Divergent Clonal Evolution of Castration-Resistant Neuroendocrine Prostate Cancer. Nat. Med..

[5] Romero R., Chu T., González Robles T.J., Smith P., Xie Y., Kaur H., Yoder S., Zhao H., Mao C., Kang W. (2024). The Neuroendocrine Transition in Prostate Cancer Is Dynamic and Dependent on ASCL1. Nat. Cancer.

[6] Rodarte K.E., Heyman S.N., Guo L., Flores L., Savage T.K., Villarreal J., Deng S., Xu L., Shah R.B., Oliver T.G. (2024). Neuroendocrine Differentiation in Prostate Cancer Requires ASCL1. Cancer Res..

[7] Puca L., Gavyert K., Sailer V., Conteduca V., Dardenne E., Sigouros M., Isse K., Kearney M., Vosoughi A., Fernandez L. (2019). Delta-like Protein 3 Expression and Therapeutic Targeting in Neuroendocrine Prostate Cancer. Sci. Transl. Med..

[8] Hanahan D. (2022). Hallmarks of Cancer: New Dimensions. Cancer Discov..

[9] Luu Hoang K.N., Anstee J.E., Arnold J.N. (2021). The Diverse Roles of Heme Oxygenase-1 in Tumor Progression. Front. Immunol..

[10] Chiang S.K., Chen S.E., Chang L.C. (2019). A Dual Role of Heme Oxygenase-1 in Cancer Cells. Int. J. Mol. Sci..

[11] Ferrando M., Gueron G., Elguero B., Giudice J., Salles A., Leskow F.C., Jares-Erijman E.A., Colombo L., Meiss R., Navone N. (2011). Heme Oxygenase 1 (HO-1) Challenges the Angiogenic Switch in Prostate Cancer. Angiogenesis.

[12] Gueron G., De Siervi A., Ferrando M., Salierno M., De Luca P., Elguero B., Meiss R., Navone N., Vazquez E.S. (2009). Critical Role of Endogenous Heme Oxygenase 1 as a Tuner of the Invasive Potential of Prostate Cancer Cells. Mol. Cancer Res..

[13] Elguero B., Gueron G., Giudice J., Toscani M.A., de Luca P., Zalazar F., Coluccio-Leskow F., Meiss R., Navone N., de Siervi A. (2012). Unveiling the Association of STAT3 and HO-1 in Prostate Cancer: Role beyond Heme Degradation. Neoplasia.

[14] Sacca P., Meiss R., Casas G., Mazza O., Calvo J.C., Navone N., Vazquez E. (2007). Nuclear Translocation of Haeme Oxygenase-1 Is Associated to Prostate Cancer. Br. J. Cancer.

[15] Lage-Vickers S., Sanchis P., Bizzotto J., Toro A., Sabater A., Lavignolle R., Anselmino N., Labanca E., Paez A., Navone N. (2022). Exploiting Interdata Relationships in Prostate Cancer Proteomes: Clinical Significance of HO-1 Interactors. Antioxidants.

[16] Scaffa A., Tollefson G.A., Yao H., Rizal S., Wallace J., Oulhen N., Carr J.F., Hegarty K., Uzun A., Dennery P.A. (2022). Identification of Heme Oxygenase-1 as a Putative DNA-Binding Protein. Antioxidants.

[17] Pamplona A., Ferreira A., Balla J., Jeney V., Balla G., Epiphanio S., Chora Â., Rodrigues C.D., Gregoire I.P., Cunha-Rodrigues M. (2007). Heme Oxygenase-1 and Carbon Monoxide Suppress the Pathogenesis of Experimental Cerebral Malaria. Nat. Med..

[18] Salloom R.J., Ahmad I.M., Sahtout D.Z., Baine M.J., Abdalla M.Y. (2024). Heme Oxygenase-1 and Prostate Cancer: Function, Regulation, and Implication in Cancer Therapy. Int. J. Mol. Sci..

[19] Yu Z., Zhu J., Wang H., Li H., Jin X. (2021). Function of BCLAF1 in Human Disease. Oncol. Lett..

[20] Norman M., Rivers C., Lee Y.B., Idris J., Uney J. (2016). The Increasing Diversity of Functions Attributed to the SAFB Family of RNA-/DNA-Binding Proteins. Biochem. J..

[21] Sun T., Li X., Zhang Y., Zou B., Zhang Y. (2024). ILF2: A Multifaceted Regulator in Malignant Tumors and Its Prospects as a Biomarker and Therapeutic Target. Front. Oncol..

[22] Li Y., Fischer P., Wang M., Zhou Q., Song A., Yuan R., Meng W., Chen F.X., Lührmann R., Lau B. (2025). Structural Insights into Spliceosome Fidelity: DHX35–GPATCH1-Mediated Rejection of Aberrant Splicing Substrates. Cell Res..

[23] Xu K., Sun S., Yan M., Cui J., Yang Y., Li W., Huang X., Dou L., Chen B., Tang W. (2022). DDX5 and DDX17—Multifaceted Proteins in the Regulation of Tumorigenesis and Tumor Progression. Front. Oncol..

[24] Jiang C., Sun L., Wen S., Tian Y., Xu C., Xu Q., Xue H. (2024). BRIX1 Promotes Ribosome Synthesis and Enhances Glycolysis by Selected Translation of GLUT1 in Colorectal Cancer. J. Gene Med..

[25] Markiewicz A., Sigorski D., Markiewicz M., Owczarczyk-Saczonek A., Placek W. (2021). Caspase-14—From Biomolecular Basics to Clinical Approach. A Review of Available Data. Int. J. Mol. Sci..

[26] Priyanka P.P., Yenugu S. (2021). Coiled-Coil Domain-Containing (CCDC) Proteins: Functional Roles in General and Male Reproductive Physiology. Reprod. Sci..

[27] Neumann C.A., Cao J., Manevich Y. (2009). Peroxiredoxin 1 and Its Role in Cell Signaling. Cell Cycle.

[28] Liu A.R., Liu Y.N., Shen S.X., Yan L.R., Lv Z., Ding H.X., Wang A., Yuan Y., Xu Q. (2022). Comprehensive Analysis and Validation of Solute Carrier Family 25 (SLC25) and Its Correlation with Immune Infiltration in Pan-Cancer. BioMed Res. Int..

[29] Anselmino N., Labanca E., Shepherd P.D.A., Dong J., Yang J., Song X., Nandakumar S., Kundra R., Lee C., Schultz N. (2024). Integrative Molecular Analyses of the MD Anderson Prostate Cancer Patient-Derived Xenograft (MDA PCa PDX) Series. Clin. Cancer Res..

[30] Spatial Transcriptomics of De Novo NEPC and ARPC—STOMICS DataBase. https://db.cngb.org/stomics/datasets/STDS0000227/summary.

[31] Xu Z., Wang W., Yang T., Li L., Ma X., Chen J., Wang J., Huang Y., Gould J., Lu H. (2024). STOmicsDB: A Comprehensive Database for Spatial Transcriptomics Data Sharing, Analysis and Visualization. Nucleic Acids Res..

[32] Watanabe R., Miura N., Kurata M., Kitazawa R., Kikugawa T., Saika T. (2023). Spatial Gene Expression Analysis Reveals Characteristic Gene Expression Patterns of De Novo Neuroendocrine Prostate Cancer Coexisting with Androgen Receptor Pathway Prostate Cancer. Int. J. Mol. Sci..

[33] Ito S., Kayukawa N., Ueda T., Taniguchi H., Morioka Y., Hongo F., Ukimura O. (2018). MRGBP Promotes AR-Mediated Transactivation of KLK3 and TMPRSS2 via Acetylation of Histone H2A.Z in Prostate Cancer Cells. Biochim. Biophys. Acta (BBA) -Gene Regul. Mech..

[34] Zhang L., Seitz L.C., Abramczyk A.M., Liu L., Chan C. (2010). CAMP Initiates Early Phase Neuron-like Morphology Changes and Late Phase Neural Differentiation in Mesenchymal Stem Cells. Cell. Mol. Life Sci..

[35] Zhang Y., Shi M., Zhang Y., Wang J., Zhang H., Ren G. (2025). RB1 and P53 Are Diagnostic Markers for Treatment-Related Neuroendocrine Prostate Cancer: A Clinical and Pathological Analysis of 23 Cases. Am. J. Clin. Exp. Urol..

[36] Paez A.V., Pallavicini C., Schuster F., Valacco M.P., Giudice J., Ortiz E.G., Anselmino N., Labanca E., Binaghi M., Salierno M. (2016). Heme Oxygenase-1 in the Forefront of a Multi-Molecular Network That Governs Cell–Cell Contacts and Filopodia-Induced Zippering in Prostate Cancer. Cell Death Dis..

[37] Loboda A., Jozkowicz A., Dulak J. (2015). HO-1/CO System in Tumor Growth, Angiogenesis and Metabolism—Targeting HO-1 as an Anti-Tumor Therapy. Vasc. Pharmacol..

[38] Nitti M., Piras S., Marinari U.M., Moretta L., Pronzato M.A., Furfaro A.L. (2017). HO-1 Induction in Cancer Progression: A Matter of Cell Adaptation. Antioxidants.

[39] Lin Q., Weis S., Yang G., Weng Y.H., Helston R., Rish K., Smith A., Bordner J., Polte T., Gaunitz F. (2007). Heme Oxygenase-1 Protein Localizes to the Nucleus and Activates Transcription Factors Important in Oxidative Stress. J. Biol. Chem..

[40] Ben-Eltriki M., Gayle E.J., Walker N., Deb S. (2023). Pharmacological Significance of Heme Oxygenase 1 in Prostate Cancer. Curr. Issues Mol. Biol..

[41] Ahanger A.A., Prawez S., Leo M.D.M., Kathirvel K., Kumar D., Tandan S.K., Malik J.K. (2010). Pro-Healing Potential of Hemin: An Inducer of Heme Oxygenase-1. Eur. J. Pharmacol..

[42] Zhang Y., Zheng D., Zhou T., Song H., Hulsurkar M., Su N., Liu Y., Wang Z., Shao L., Ittmann M. (2018). Androgen Deprivation Promotes Neuroendocrine Differentiation and Angiogenesis Through CREB-EZH2-TSP1 Pathway in Prostate Cancers. Nat. Commun..

[43] Anselmino N., Bizzotto J., Sanchis P., Lage-Vickers S., Ortiz E., Valacco P., Paez A., Labanca E., Meiss R., Navone N. (2020). HO-1 Interactors Involved in the Colonization of the Bone Niche: Role of ANXA2 in Prostate Cancer Progression. Biomolecules.

[44] Krämer A., Green J., Pollard J., Tugendreich S. (2014). Causal Analysis Approaches in Ingenuity Pathway Analysis. Bioinformatics.

[45] Wu T., Hu E., Xu S., Chen M., Guo P., Dai Z., Feng T., Zhou L., Tang W., Zhan L. (2021). ClusterProfiler 4.0: A Universal Enrichment Tool for Interpreting Omics Data. Innovation.

[46] Bioconductor—Enrichplot. https://bioconductor.org/packages/release/bioc/html/enrichplot.html.

[47] Cerami E., Gao J., Dogrusoz U., Gross B.E., Sumer S.O., Aksoy B.A., Jacobsen A., Byrne C.J., Heuer M.L., Larsson E. (2012). The CBio Cancer Genomics Portal: An Open Platform for Exploring Multidimensional Cancer Genomics Data. Cancer Discov..

[48] Gao J., Aksoy B.A., Dogrusoz U., Dresdner G., Gross B., Sumer S.O., Sun Y., Jacobsen A., Sinha R., Larsson E. (2013). Integrative Analysis of Complex Cancer Genomics and Clinical Profiles Using the CBioPortal. Sci. Signal..

[49] De Bruijn I., Kundra R., Mastrogiacomo B., Tran T.N., Sikina L., Mazor T., Li X., Ochoa A., Zhao G., Lai B. (2023). Analysis and Visualization of Longitudinal Genomic and Clinical Data from the AACR Project GENIE Biopharma Collaborative in CBioPortal. Cancer Res..

[50] Kolde R. (2025). R Package Pheatmap.

[51] Kassambara A., Mundt F. (2026). R Package Factoextra.

[52] Livak K.J., Schmittgen T.D. (2001). Analysis of Relative Gene Expression Data Using Real-Time Quantitative PCR and the 2^−ΔΔCT^ Method. Methods.

[53] Wickham H. (2016). ggplot2.

[54] Kassambara A. (2026). R Package.

[55] Neuwirth E. (2022). R Package RColorBrewer.

