# Supplementary material for: HO-1 Nuclear Interactome Implications in Neuroendocrine Transdifferentiation in Prostate Cancer
Source: Int J Mol Sci. 2026 Aug 26;27(17):7635. doi: 10.3390/ijms27177635 (PMC13566780; doi:10.3390/ijms27177635)
Supplement: Supplementary file 1 [file ijms-27-07635-s001.zip › ijms-4371314-supplementary.pdf]

Supplementary Table S1. HO-1 nuclear interactors' target genes and binding scores

SAFB TARGET GENES (655)					
TARGET GENE	BINDING SCORE	TARGET GENE	BINDING SCORE	TARGET GENE	BINDING SCORE
TDRD7	2854	CALR	1702.333333	AP4E1	1346
TMCO3	2634.333333	MFSD14A	1684.333333	RGS10	1337
DCUN1D2	2634.333333	RAPGEF6	1680.666667	FAM227B	1321.666667
RAB14	2629.666667	LRP2BP	1680.666667	DTWD1	1321.666667
GSN	2629.666667	ANKRD37	1680.666667	RABGAP1L	1318.333333
GOLGA7	2523	RPAP3	1679.333333	RTL10	1307.666667
ZNF558	2502.666667	MTDH	1673.333333	GNB1L	1307.666667
TEAD1	2499.666667	IMMP2L	1658.333333	NRARP	1300
ZNHIT2	2477	TIPARP	1652	FBXO4	1293.666667
TM7SF2	2477	EIF4ENIF1	1639.666667	SEPTIN5	1290
GATAD2A	2467	IDUA	1639.333333	PPDPF	1289.666667
PIEZO1	2428.666667	YTHDF2	1633.666667	PAXX	1289
RING1	2391	H6PD	1632.333333	CLIC3	1289
HSD17B8	2391	CUL9	1627.333333	HAGHL	1286
UBE2I	2378.666667	NBEAL2	1626.666667	CCDC78	1286
DNAJC5	2372.666667	CCDC12	1626.666667	TUBB4B	1281
ZBTB7B	2355	ZC3H4	1620	TMEM248	1280.666667
RNF187	2351.666667	PRRC2B	1615.333333	VGLL4	1276
FBRS	2323.333333	CERCAM	1603.333333	CAPN15	1270.666667
CAMTA1	2279.333333	H2BU1	1599.666667	FRYL	1263.333333
ZNF581	2219	H2AW	1599.666667	JUND	1262
CCDC106	2219	JADE1	1595.666667	APRT	1257
KXD1	2205.333333	ESYT2	1595.666667	TLK2	1252.333333
ZNF555	2182.666667	ANKRD12	1595.666667	SLC10A7	1247
CDT1	2143.333333	D2HGDH	1594.333333	RUBCN	1238
CDKN1A	2139.666667	UNKL	1576	FYTTD1	1238
HIBADH	2139	P4HA1	1568.666667	SUV39H1	1224.666667
GLI4	2130	SEC24B	1554	RTN4	1221.333333
VPS9D1	2081.333333	SUN2	1545.333333	RGL1	1211.666667
DNPEP	2079.666667	TIAM2	1544.333333	ARPC5	1211.666667
ANKRD31	2057.333333	RASGRP1	1541.666667	CREG1	1206
NOTUM	2040.666667	TLCD1	1523.666667	KLHL36	1203
QKI	2007.666667	NEK8	1523.666667	ZNF696	1202
THAP9	1993.666667	THAP7	1494	PPP1CB	1201
SEC31A	1993.666667	RBM4B	1488.333333	HEXIM1	1194
BEND3	1962.666667	JAGN1	1479	S1PR2	1192.666667
SCAF1	1951.333333	TMEM214	1478	GTF2I	1191.333333
RRAS	1951.333333	FBXO48	1477.333333	PRMT2	1188.333333
KCTD18	1950	APLF	1477.333333	CAPRIN1	1179.333333
TTLL4	1940	EBAG9	1464.666667	SLC25A40	1177.666667
PSEN2	1903.666667	MORF4L2	1453.333333	DBF4	1177.666667
TXLNA	1895.666667	ARPC2	1451	ANXA5	1175.666667
NSUN4	1880.666667	SLC16A1	1447.666667	FBXL20	1170.333333
ARHGDIA	1868.666667	ASXL1	1441	SPART	1169
ZNF77	1866	MRPL40	1437.666667	CIRBP	1166.333333
SYPL1	1848.333333	HIRA	1437.666667	PCM1	1165
PGAM5	1837.333333	IVNS1ABP	1434.666667	TXNDC9	1160.333333
NFIB	1817.333333	AHCYL2	1414.666667	EIF5B	1160.333333
RNASEK	1814	PABPC4	1409.333333	TAMM41	1157.333333
C17orf49	1814	SKA3	1401.666667	KIAA1586	1156.333333
RAP2B	1801.333333	MRPL57	1401.666667	TIMM17A	1138.666667
NOP53	1783.333333	USP54	1393.333333	TRAPPC5	1137
KMT5B	1780.333333	SLC25A45	1388	MCEMP1	1137
ATAD3A	1767.333333	FRMD8	1388	RNASEH2B	1120.666667
CCNL1	1761.333333	CLK3	1383.666667	MLST8	1113.666667
TCF4	1750.333333	POLR3H	1383	SHC2	1112
ATP5F1E	1748.333333	PTBP1	1377.666667	MMAA	1110.333333
SLC35A4	1726.333333	TNFRSF18	1374.333333	IRF1	1103

APBB3	1726.333333	NECAB2	1371.333333	PRAME	1102.666667
ZC3H6	1724.333333	FH	1362.666667	LRRC42	1102.333333
FARSA	1702.333333	CCNY	1350.666667	HSPB11	1102.333333
CYREN	1099	LIME1	970	RNF157	847.333333
DENND4A	1098.666667	KMT2C	968.666667	NFIC	846.666667
CEP83	1095	CSNK1E	966.666667	MTPAP	845
ASB6	1092.333333	DHDDS	965.333333	PFN1	838
ATF6	1090.666667	CCDC116	962	ENO3	838
RNF207	1090.333333	TRIM24	960.333333	RHEB	836.333333
TBPL1	1086.666667	RAB3A	960.333333	TBC1D14	834
MYLIP	1083.666667	MAP3K20	958.666667	NR3C1	834
TMEM249	1083	BAIAP3	957.666667	ZBTB12	832
SLC52A2	1083	WTAP	955.666667	EHMT2	832
FBXL6	1083	SOD2	955.666667	C2	832
XYLB	1081	CTBP2	953.333333	HK2	828.333333
FAHD2A	1076.666667	SLAIN2	949.666667	RAB11FIP4	825.666667
DEK	1076.333333	TCP11L2	948	OTULIN	822.333333
CCDC91	1075.333333	SMIM30	947	JARID2	821
SAPCD2	1075	PPP1R13L	946.333333	MAT2A	820
MRI1	1071	POLR1G	946.333333	DLX2	819.666667
STIM2	1069.666667	NFKBID	946.333333	BRK1	819.333333
MKLN1	1067.666667	HCST	946.333333	RANBP9	814.666667
ZNF384	1066	TMED7-TICAM2	945.666667	NAGPA	813.333333
JAK2	1061	TMED7	945.666667	BTG2	812.666667
CORO7-PAM16	1060.666667	SMYD5	943.666667	CYP4V2	811.333333
CORO7	1060.666667	DDHD2	940.666667	KCNH4	811
STUB1	1059	COMMD7	938.333333	HCRT	811
RHBDL1	1059	PHLDB1	935	QSER1	806.666667
TICAM1	1057.333333	BCAR3	932.666667	ANTXR1	804.333333
PLCG1	1057	HUS1	931.666667	RCOR1	804
ASF1A	1052.333333	MIPOL1	929.333333	ASMTL	798.333333
DAGLA	1051.666667	HS3ST6	928.666667	KCNK5	797.333333
RAP1GAP	1040	RCC2	926.666667	DISP3	797.333333
CDK6	1038.666667	SLC1A4	924.666667	PRICKLE3	792
TYMP	1038.333333	OIP5	919.666667	ATAD3B	792
SCO2	1038.333333	NUSAP1	919.666667	FHDC1	789.666667
DGKQ	1037.333333	ODF3L2	915.666667	FUT11	789
WASF1	1035.333333	TMEM170B	913.666667	NME2	784.333333
CDC40	1035.333333	CD300LG	912.333333	PYCR3	782.333333
ACHE	1035	GPR158	910.666667	LTB	779
KIFC3	1032	VEGFA	910	STK17A	776.333333
ACTN1	1030.666667	SERPINB6	907.333333	UBE2N	773.333333
NSD3	1028.666667	NADK2	906.333333	NUDCD2	771
LETM2	1028.666667	COTL1	906.333333	HMMR	771
ZNF185	1028	MAFK	905.333333	ARRB1	768.333333
PFN3	1025	HP1BP3	905.333333	H4C2	767.333333
F12	1025	ZNF584	903	H3C2	767.333333
ZNF57	1023.333333	DSCC1	902	H2AC4	767.333333
ZNF316	1021	RNF170	897	YPEL2	765
H3C15	1019	RHOBTB1	897	ICE2	764.666667
H3C14	1019	HOOK3	897	PKMYT1	764.333333
H2AC19	1019	PPFIA3	890.666667	ZNF263	761
H2AC18	1019	LIN7B	890.666667	SZRD1	760.666667
ZNF236	1011.333333	C19orf73	890.666667	CHCHD3	755
LRWD1	1011	TUBB	885	DNAJC11	752
ALKBH4	1011	MDC1	885	LRP8	750.666667
POLE2	1009	LEF1	868.666667	SDHB	748.666667
KLHDC1	1009	MFSD3	868.333333	PCNA	747.333333
CORO1A	1008.333333	C8orf37	864.333333	CDS2	747.333333
PPP1R18	1003	AEBP2	864	SFI1	745
NRM	1003	CCDC57	862.666667	TIRAP	744.666667
TTC3	986.333333	WRNIP1	858.666667	COLGALT1	744.666667
PIGP	986.333333	MYLK4	858.666667	ANP32B	744.666667

KHDRBS1	985.666667	KLF15	854	GALNT11	742.333333
FAM120B	972.666667	NOM1	851.666667	PFN4	741
SLC2A4RG	970	ZP3	850.333333	FAM228B	741
TXNRD2	740.666667	ASPDH	673	DDX19B	605
COMT	740.666667	NRROS	672.333333	PLD6	604.666667
MAPK6	740.333333	ZMIZ1	672	SLK	604.333333
CCSAP	739.666667	WDR45	672	NFE2L2	604.333333
ACSF3	739	FAM156B	671.666667	CBLL1	603.666667
SPTBN4	738.666667	FAM156A	671.666667	ACTR10	602.666667
BLVRB	738.666667	KLF3	671.333333	RAB6A	600.333333
YIF1A	737.333333	PPARGC1B	670.666667	GULP1	598.333333
TMEM151A	737.333333	UHRF1	666	ATG5	598.333333
AKAP1	737.333333	DYRK2	665	SPCS3	598
RGS20	736.333333	LPCAT4	659.333333	ING1	596.666667
LMBR1	733.333333	PRSS21	658.666667	RNF44	595.666667
SGSM3	730.666667	FECH	654.333333	PRADC1	595.333333
ABCC5	729.333333	ZBTB47	653.333333	CCT7	595.333333
C11orf96	729	USP4	652.333333	RNF145	595
MYADM	727.333333	LRRC24	652.333333	PGAP6	595
PMF1-BGLAP	726	C8orf82	652.333333	SNX25	594.333333
PMF1	726	KDM6B	650.333333	PPP1R27	594.333333
MAP2K5	725	GAA	650.333333	MCRIP1	594.333333
SLC4A7	724	KHK	647.666667	CFAP97	594.333333
KIAA0895	721	FLCN	647	JMJD7-PLA2G4B	593
DNAH14	720.666667	CBSL	647	JMJD7	593
MATN1	719.333333	CBS	647	KCTD15	592.333333
CCDC97	719	STXBP3	646.666667	MECP2	591.333333
ZNF839	718.666667	BMP2K	645.666667	ADARB1	590
TMEM267	718.333333	CAND1	642.333333	ZNF721	589
PIGW	716.666667	MUC12	638	PIGG	589
MYO19	716.666667	NRIP3	636.666667	CEP295	588
UBE2D3	715.333333	SHQ1	636.333333	TAF4B	587.333333
MAP1LC3B	714.666667	ZNF596	635.333333	SLC27A1	587
FBXO31	714.666667	TMEM64	634.666667	CTF1	584.666667
HNRNPA2B1	713	MIF4GD	633	BCL7C	584.666667
CBX3	713	UBE2R2	632.666667	APOLD1	584
TRAP1	712	IQGAP2	630	TWF2	583.666667
RSBN1L	711	PEBP1	629.666667	MXI1	582.666667
SLC7A5	709	H3C4	629	CSNK1G2	582.333333
TUT4	708.333333	H2BC7	629	PXT1	581.666667
FUNDC2	708	H2AC7	629	KCTD20	581.666667
F8	708	TEX15	628	PIGF	578.333333
PIGX	707.666667	KLF10	628	CRIPT	578.333333
CEP19	707.666667	PRSS27	627.666667	TBCD	578
RASIP1	701	ZFP1	623.333333	TRAF2	577.333333
SEC63	697.666667	C5orf24	621.333333	JMJD8	575.333333
PHOSPHO2	697.666667	DAZAP1	620.333333	TEX46	575
MAN2C1	697.666667	NRL	616.666667	KDM1A	575
CCDC173	697.666667	DCAF11	616.666667	CALU	574
ZDHHC14	697	PAXIP1	615.666667	SAR1A	573.333333
SLC38A2	693.333333	SETD5	615.333333	JPT2	573.333333
LRRC59	692.333333	ARID2	613.666667	HIP1	571.666667
SMC5	691.333333	ZNF783	613	GTF3C4	571.666667
HMGA1	691.333333	RIPK1	611.666667	DDX31	571.666667
GGH	691	HERC2	610	ZFP36	569.666667
EZH2	690	PRDM15	609.333333	POLM	569.666667
MIDN	687.333333	NKD2	609	BTBD10	568
MORC1	685.333333	OTUD1	608.333333	RTKN	565.333333
FNIP2	683	KIAA1191	608	GPAT4	564.666667
ADSS1	678.666667	ARL10	608	TMEM161B	564
MTHFD2L	677.666667	TEX264	607.333333	SSBP4	564
PLEKHA3	677	IER5L	607.333333	SAMD13	564
FKBP7	677	APBB1	607	IGF2BP3	563.666667

SYNJ2	673.666667	ZNF546	606.666667	TMEM70	561
DTD1	673.666667	SCLY	606	ELOC	561
JOSD2	673	MTF1	605.333333	GDPGP1	559.666667
CIB1	559.666667	RHOA	538.333333	MROH1	513.666667
TBC1D24	558.666667	C2orf42	538	RPS19	513.333333
NTN3	558.666667	NCAM2	537.666667	RFX3	513
ZNF800	557.333333	KIFC2	537.333333	PGGHG	513
UBALD1	554.666667	CYHR1	537.333333	GALE	513
GSE1	554.666667	GPX4	536.333333	SNX17	512
ATL2	552.666667	CNDP2	536.333333	EIF2B4	512
SMCHD1	551.666667	ARL8B	536.333333	TBCCD1	511.666667
MACROD1	551	CARMIL3	534	DNAJB11	511.666667
SS18L1	550.666667	ZER1	533.666667	CSK	511.666667
PSMA7	550.666667	CAPN7	533.333333	AREG	510.666667
SLC45A3	550	AGBL3	532.333333	RCAN1	509
GCLC	549.666667	CELF6	531	POU3F1	509
SCRN2	549.333333	DIAPH1	528	PIP5K1A	507
ATXN2L	549.333333	NAPEPLD	527.666667	ST3GAL3	505.666667
ICAM5	548.666667	FOSL1	527.666667	CMTM3	505.666667
ICAM4	548.666667	TMEM97	526.666667	ITGB1BP1	505.333333
ZNF697	547.666667	PXK	526.666667	CPSF3	505.333333
SLC25A24	547.666667	PLOD1	524.333333	CDK17	504.666667
CMBL	547	INSIG1	523.666667	CBLB	504
TIGD2	546.666667	CTCF	522.333333	TRIM45	503
SH3GL3	546.666667	CACNA1H	521.333333	STPG3	502.333333
DNAAF2	545.666667	ZNF891	521	NELFB	502.333333
L3HYPDH	545.333333	ZNF10	521	CNP	502.333333
JKAMP	545.333333	ST6GALNAC6	520.333333	NEK6	501.333333
HNRNPH1	545.333333	CIC	520.333333	NEIL3	501.333333
DENND6B	545.333333	NRN1	519	IQSEC3	501.333333
NFKB1	543.666667	ZNRF2	516.333333	TMUB1	500.333333
LUC7L2	540.333333	RPRD1A	516.333333	FASTK	500.333333
SLBP	539.666667	SOS2	514.333333	AGAP3	500.333333
MAN1B1	539.666667	EEF1A2	514.333333		
TCTA	538.333333	SCRT1	513.666667		

BCLAF1 TARGET GENES (118)

TARGET GENE	BINDING SCORE	TARGET GENE	BINDING SCORE	TARGET GENE	BINDING SCORE
CDIPT	984.2	CDON	767.4	ARPC4	685.7
ATG101	960.7	EED	757.8	NR6A1	685.2
MTRNR2L1	891.3	FOS	748.9	GNB2	679.3
PIK3R3	874.5	HDAC2	743.8	BCL6	674.9
CENPN	845.1	C21orf91	736.6	PRKCI	671.1
DOHH	828.5	C6orf62	722.4	ATP8A1	670.4
GIT2	807.8	LRPPRC	721.9	CD164	668.9
ANKRD13A	807.8	WASF2	715.3	PPP1R13L	668.8
FUS	802.7	ALDH4A1	711.8	POLR1G	668.8
TARDBP	802.2	PSIP1	706.8	SLC35E2B	666
TIGD5	799.5	PTDSS1	704.7	EIF3B	665.8
EEF1D	799.5	MTERF3	704.7	PARK7	659.4
SLC35E2A	786.5	DGAT2	703.6	HYAL2	651.5
FLCN	781	TCP11L2	701.3	FAM53C	649.4
NOL8	779.9	UFD1	700.1	CDC25C	649.4
CENPP	779.9	CDC45	700.1	ATF7-NPFF	646.5
UROS	779.3	NOCT	699.1	ATF7	646.5
BCCIP	779.3	ZNF219	698.7	SELENOW	635.8
YY1AP1	778.8	TMEM253	698.7	RPN2	633.5
DAP3	778.8	TADA3	685.7	MROH8	633.5
PDXDC1	767.8	ARPC4-TTLL3	685.7	MYL12A	628.1
UQCRC2	615.6	CALM3	575.5	TAF15	538
FBXO33	614	UQCRH	572	RPL19	537.3
MYO9B	611.1	LRRC41	572	ULK4	536.7
HAUS8	611.1	DPP9	569.6	PFDN4	534.3

CDCA7L	609	FRG2C	566.6	MYL12B	533.6
UBE2B	604.1	TRIP10	563.3	ASH1L	532.3
CDKL3	604.1	GPR108	563.3	FRAT1	530.1
PMVK	603.2	ZFAND3	562.8	CACNB1	528
UFSP2	600.3	UTP18	561.3	FAM227A	527
C4orf47	600.3	MBTD1	561.3	CBY1	527
SMC3	597.7	POLG	557.7	TMEM260	525.5
PRMT1	593.6	TXNIP	556.7	COMMD6	524.1
PHRF1	590.6	NDUFB1	555.9	FKBP3	522.4
SS18	588.6	CPSF2	555.9	FANCM	522.4
LSR	585.9	PAXIP1	547.5	STK35	519.2
RHBDD3	585.5	KCNAB2	546.3	SNAP29	506.7
EWSR1	585.5	IQCH	540.5	PI4KA	506.7
SIRT4	585.3	AAGAB	540.5		
PPP5D1	575.5	POR	539.8		

DDX17 TARGET GENES (17)

TARGET GENE	BINDING SCORE	TARGET GENE	BINDING SCORE	TARGET GENE	BINDING SCORE
RAPGEF4	618	HCK	579.666667	CNOT6	522.166667
C1orf198	613.333333	TAX1BP1	554.166667	NGF	520.666667
SLC22A16	609	HLA-F	551.166667	PEX12	509.5
MIB1	598.833333	TCP11L1	535.166667	AP2B1	509.5
TMSB4Y	596.5	TAPT1	526.666667	CCDC112	502.5
INKA2	596.5	HIPK3	522.833333		

ILF3 TARGET GENES (8)

TARGET GENE	BINDING SCORE	TARGET GENE	BINDING SCORE	TARGET GENE	BINDING SCORE
MTRNR2L1	1174.666667	FRG2C	815.666667	DDAH2	567.666667
WDR74	1152	SIRT4	789.666667	LZTR1	535.333333
DNAJB4	1127.333333	TMEM107	750.666667		

VALIDATION OF PROTEIN-PROTEIN INTERACTIONS THROUGH IMMUNOPRECIPITATION FOLLOWED BY WESTERN BLOT

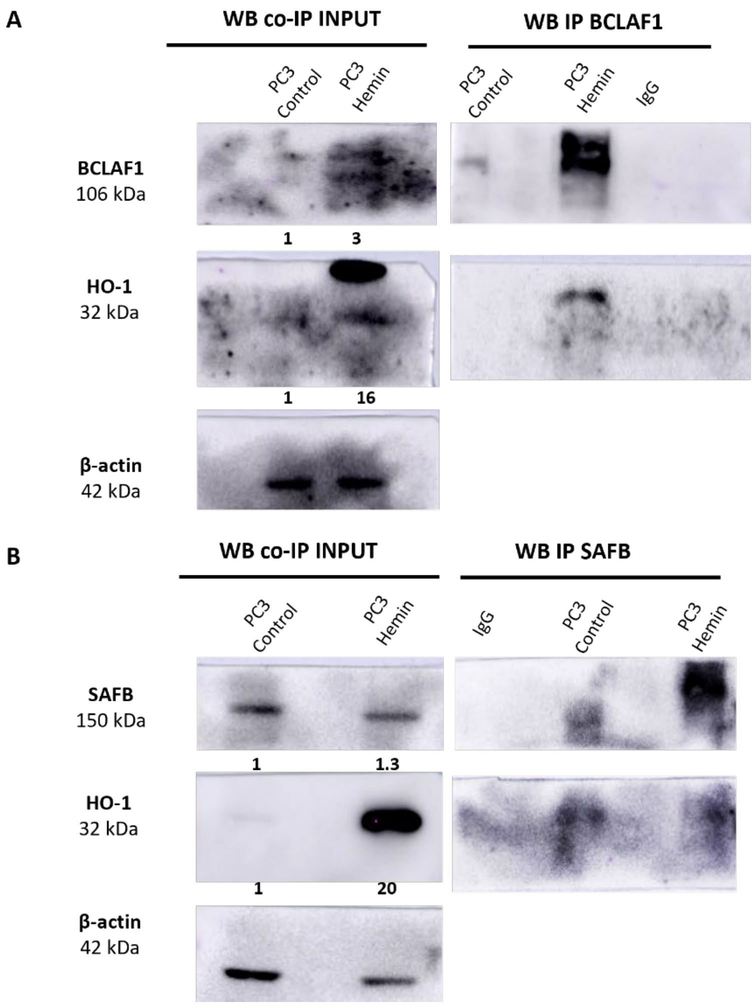

Supplementary Figure S1. Validation of protein-protein interactions through immunoprecipitation followed by western blot. *Co-IP analysis of BCLAF1* ***(A)*** *and SAFB* ***(B)*** *in PC3 cells treated with 80 μM hemin for 24 h. IgG was used as specificity control. Coprecipitated proteins were identified by western blotting, and we used Actin as loading control. Quantification values below each blot represent normalized expression relative to the untreated control.*

PRINCIPAL COMPONENT ANALYSIS POINTS TO ILF3 AS THE STRONGEST CONTRIBUTOR TO NEPC-ADENO VARIANCE

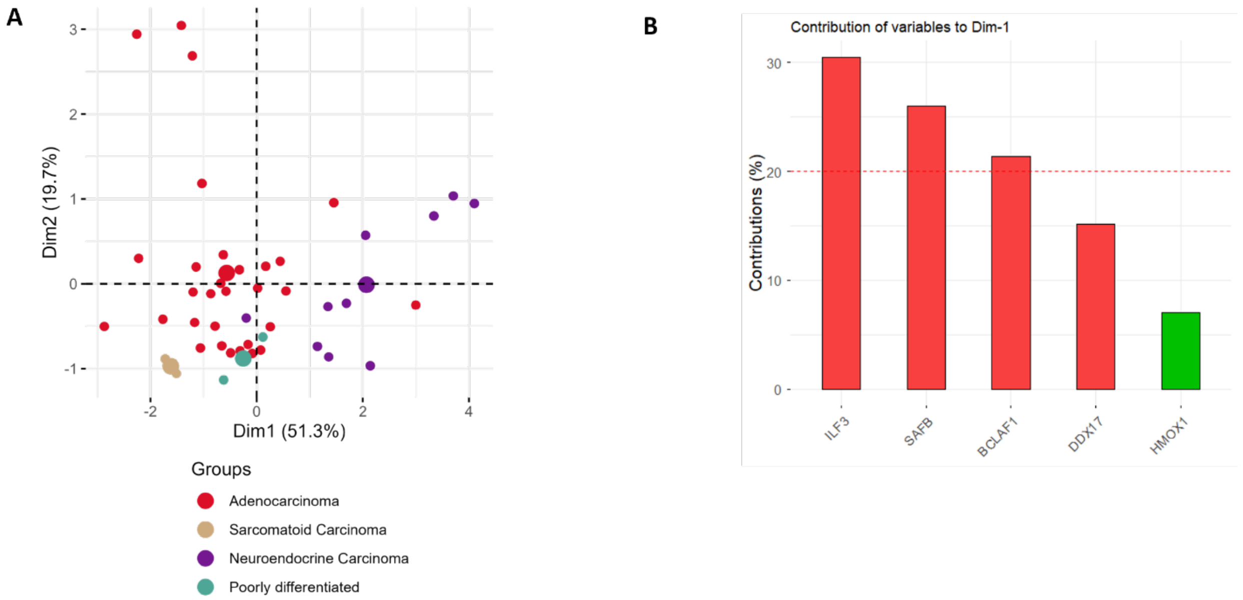

Supplementary Figure S2. ILF3 is a major contributor to the NEPC-Adeno variance. (A) *Principal Component Analysis plot from the unsupervised clustering analysis of RNAseq data from the 44 MDA PCa PDXs considering the expression of HMOX1, ILF3, SAFB, BCLAF1 and DDX17. Each point represents one PDX. Samples are colored according to the histopathological classification: adenocarcinoma (red), sarcomatoid (beige), neuroendocrine (purple) and poorly differentiated (green).* ***(B)*** *Bar graph represents the contribution (%) of each gene to the variance between samples. The red dashed line depicts the expected average contribution if all genes weighed the same (value = 20%).*

COMPARISON OF PDXS FROM THE SAME TUMOR WITH DIFFERENT HISTOPATHOLOGICAL FEATURES REVEALED HIGH EXPRESSION OF HO-1 INTERACTORS IN NEPC PDX 146-10

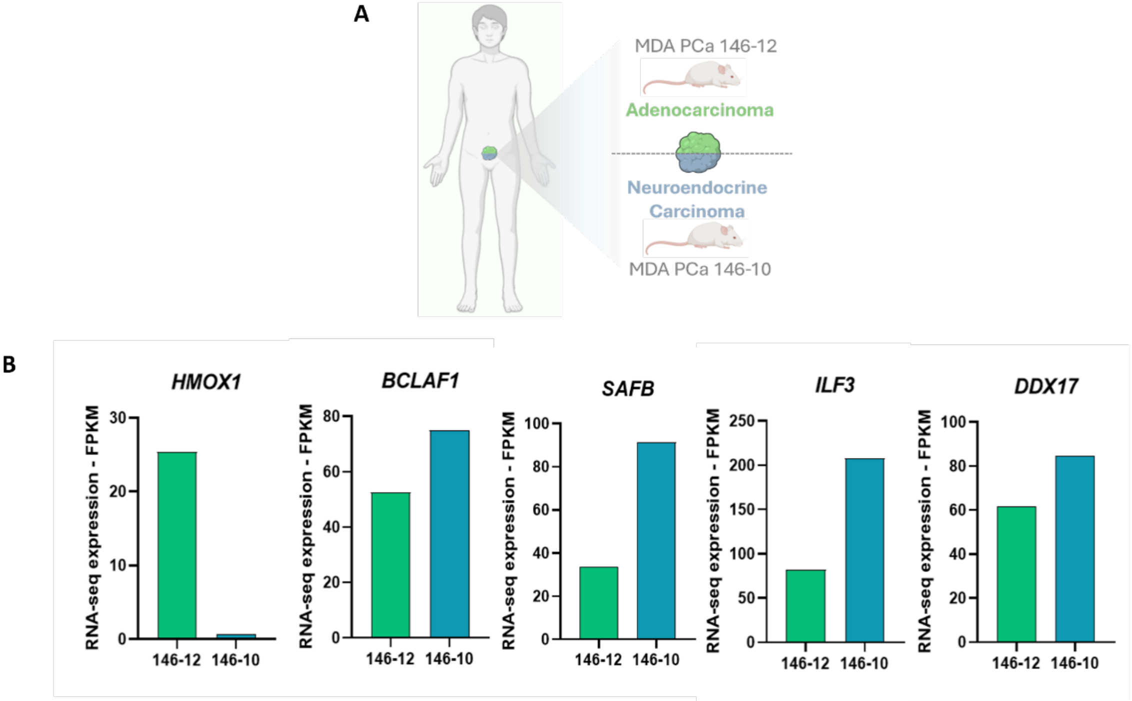

Supplementary Figure S3. Comparison of gene expression (FPKM) of HMOX1, BCLAF1, SAFB, ILF3, and DDX17 between the PDXs MDA PCa 146-12 (adenocarcinoma) and MDA PCa 146-10 (NEPC), generated from the same prostate tumor. (A) *Schematic representation of the generation of the PDX 146-10 and PDX 146-12, subsequently sequenced.* ***(B)*** *Bar graphs showing the expression levels (FPKM) of HMOX1, BCLAF1, SAFB, ILF3, and DDX17, determined from an RNA-seq analysis (n=1) in PDX MDA PCa 146-12 (adenocarcinoma) vs. PDX MDA PCa 146-10 (NE).*

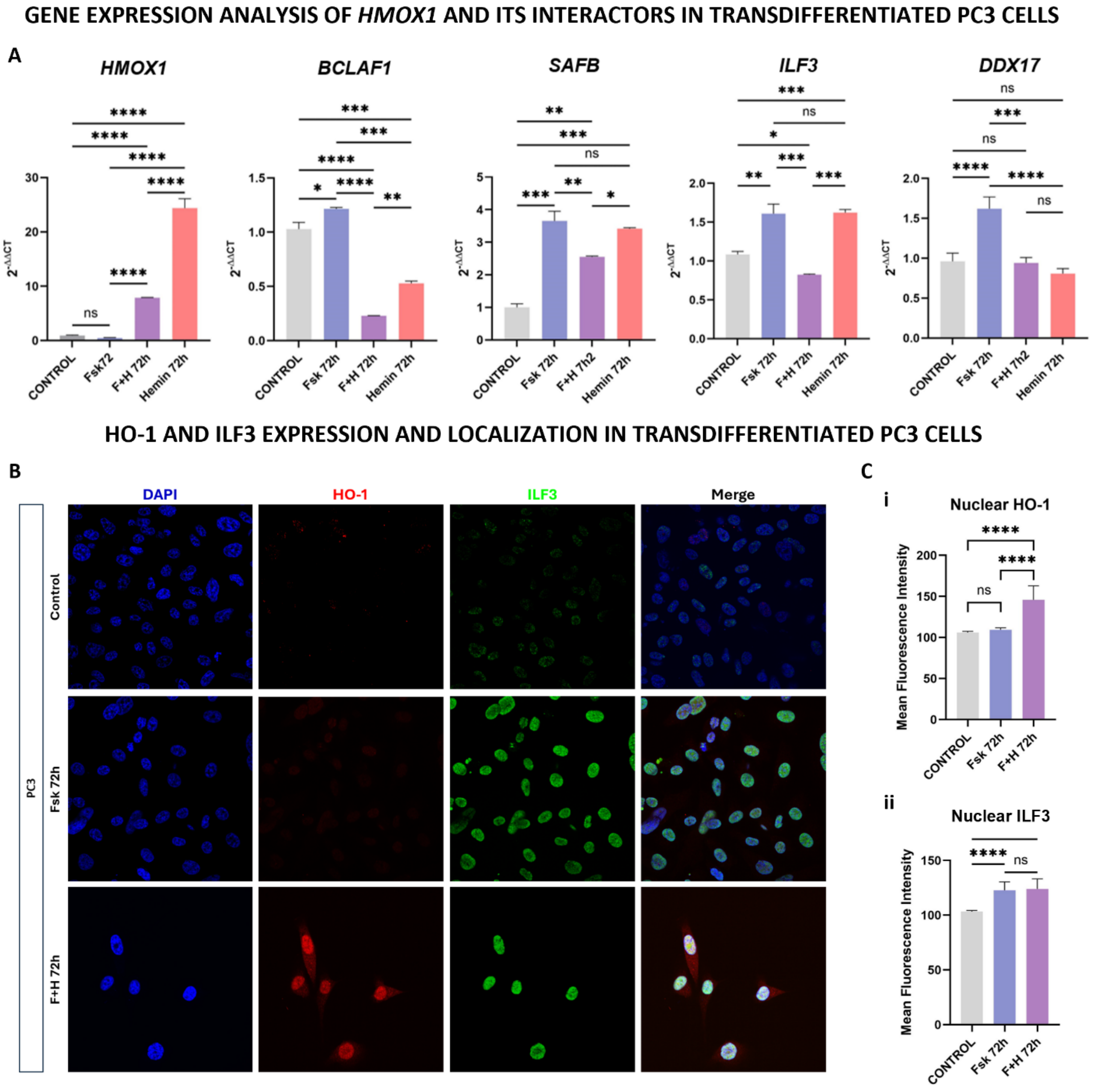

Supplementary Figure S4. Expression of HMOX1 and its nuclear interactors during fsk-induced neuroendocrine transdifferentiation and HO-1 induction. (A) *Transcript levels of HMOX1, BCLAF1, SAFB, ILF3, and DDX17 were measured by RT-qPCR in PC3 cells treated with 10 µM Fsk, 80 µM hemin or co-treated with both drugs (F+H; 10µM and 80µM) for 72 h. Fsk-induced transdifferentiation increased the expression of BCLAF1, SAFB, ILF3, and DDX17, whereas HMOX1 expression remained unchanged. Co-treatment with hemin markedly induced HMOX1 and*

attenuated the Fsk-associated increase in the four HO-1 nuclear interactors. GAPDH was used as the reference gene. ***(B)*** *Immunofluorescence staining and confocal microscopy analysis for HO-1 and ILF3 on PC3 cells treated with 10µM Fsk, co-treated with Fsk and hemin (F+H; 10µM and 80µM) or untreated (CONTROL) for 72h. Cell nuclei were counterstained with DAPI.* ***(C)*** *Quantitative analysis of mean fluorescence intensity for* ***(Ci)*** *nuclear HO-1 and* ***(Cii)*** *nuclear ILF3 was determined using FiJi. Results are presented as mean ± S.E.M. Statistical significance was set at $p < 0.05$, with *$p < 0.05$; **$p < 0.01$; ***$p < 0.001$; ****$p < 0.0001$.*
